# Methanol chemoreceptor MtpA- and flagellin protein FliC-dependent methylotaxis contributes to the spatial colonization of PPFM in the phyllosphere

**DOI:** 10.1093/ismeco/ycaf092

**Published:** 2025-05-29

**Authors:** Shiori Katayama, Kosuke Shiraishi, Kanae Kaji, Kazuya Kawabata, Naoki Tamura, Akio Tani, Hiroya Yurimoto, Yasuyoshi Sakai

**Affiliations:** Graduate School of Agriculture, Kyoto University, Kitashirakawa-Oiwake, Sakyo-ku, Kyoto 606-8502, Japan; Graduate School of Agriculture, Kyoto University, Kitashirakawa-Oiwake, Sakyo-ku, Kyoto 606-8502, Japan; Graduate School of Agriculture, Kyoto University, Kitashirakawa-Oiwake, Sakyo-ku, Kyoto 606-8502, Japan; Graduate School of Agriculture, Kyoto University, Kitashirakawa-Oiwake, Sakyo-ku, Kyoto 606-8502, Japan; Department of Anatomy and Histology, School of Medicine, Fukushima Medical University, Hikarigaoka 1, Fukushima 960-1295, Japan; Institute of Plant Science and Resources, Okayama University, 2-20-1, Chuo, Kurashiki, Okayama 710-0046, Japan; Graduate School of Agriculture, Kyoto University, Kitashirakawa-Oiwake, Sakyo-ku, Kyoto 606-8502, Japan; Graduate School of Agriculture, Kyoto University, Kitashirakawa-Oiwake, Sakyo-ku, Kyoto 606-8502, Japan; Graduate School of Advanced Integrated Studies in Human Survivability, Kyoto University, Yoshida Nakaadachi-Cho 1, Sakyo-ku, Kyoto 606-8306, Japan; Department of Bioscience and Biotechnology, Faculty of Bioenvironmental Sciences, Kyoto University of Advanced Science, 1-1 Sogabe-cho Nanjo Otani, Kameoka, Kyoto 621-0023, Japan

**Keywords:** PPFM, methylotaxis, phyllosphere, fluorescence imaging, bacterial behavior, plant-microbe interaction

## Abstract

Pink-pigmented facultative methylotrophs (PPFMs) capable of growth on methanol are dominant and versatile phyllosphere bacteria that provide positive effects on plant growth through symbiosis. However, the spatial behavior of PPFMs on plant surfaces and its molecular basis are unknown. Here, we show that *Methylobacterium* sp. strain OR01 inoculated onto red perilla seeds colonized across the entire plant surface in the phyllosphere concomitant with the plant growth. During its transmission, strain OR01 was found to be present on the entire leaf surface with a preference to sites around the periphery, vein, trichome, and stomata. We found that methanol-sensing chemoreceptor MtpA-dependent chemotaxis (methylotaxis; chemotaxis toward methanol) and flagellin protein FliC-dependent motility facilitated the bacterial entry into the stomatal cavity and their colonization in the phyllosphere.

## Introduction

*Methylobacterium* sp., referred to as pink-pigmented facultative methylotroph (PPFM), is one of the most ubiquitous and abundant bacterial genera present in the aerial parts of the plant, the phyllosphere. PPFM is estimated to exist at 10^4^–10^7^ colony forming units (CFU) per gram fresh weight of plant materials [1]. Many strains of *Methylobacterium* are known for their ability to promote plant growth through the production of plant hormones such as auxins and cytokinin, and to induce systemic resistance to pathogens and diseases [2–7]. In turn, they obtain nutrients from the host plant, establishing the symbiotic relationship in the phyllosphere.

*Methylobacterium* sp. strain OR01 is widely observed on red perilla seeds and leaves collected from various regions of Japan [8], indicating that this species is a major colonizer of red perilla leaves. Such species-level specificity of the interaction between the red perilla plant and strain OR01 makes them an ideal system for investigating the ecology and physiology of PPFMs in the phyllosphere. The factors and features that contribute to strain OR01 attaining the dominant colonizer status of perilla phyllosphere still remain unknown and need to be elucidated.

Even though the phyllosphere is speculated to be nutrient-poor, plants indeed provide nutrient compounds sufficient to sustain large microbial communities [9]. In particular, methanol, produced from the cell wall component pectin, is recognized as a relatively abundant carbon source for methanol-utilizing microorganisms [10], which proves advantageous for PPFMs and allows them to dominate the phyllosphere [11]. PPFMs have developed several survival strategies to help them adapt to the harsh and ever-changing phyllosphere environment, brought about by various factors such as UV, temperature, osmotic and oxidative stresses [12]. Previously, we revealed a periodic fluctuation in methanol concentration on *Arabidopsis* leaves, i.e. high in the dark period and low in the light period, using a yeast methanol sensor that could directly measure methanol concentrations [13]. We also showed that in response to these diurnal changes in methanol concentration, the methanol-utilizing yeast *Candida boidinii* regulates its gene expression and peroxisome homeostasis necessary for methanol metabolism.

Chemotaxis, the movement of bacteria as a response to chemical stimuli, is driven by three main components: methyl-accepting chemotaxis proteins (MCPs) as sensor chemoreceptors, Che proteins as transmitters of chemotactic signals through phosphorylation, and flagella as the driving force of directed movement [14, 15]. Our recent study on *Methylobacterium aquaticum* strain 22A showed that three MCPs (MtpA, MtpB, and MtpC) were responsible for chemotaxis toward methanol, methylotaxis [16]. A triple *M. aquaticum* strain 22A mutant of these MCPs lost methylotaxis and showed less efficient colonization on plants than the wild-type strain. The role of motility and chemotaxis in the colonization and distribution in the phyllosphere has also been investigated in pathogenic bacteria. In *Pseudomonas syringae*, the lack of CheA and CheY2 led to the loss of motility and chemotaxis, and the inability to cause severe disease symptoms on the host tobacco leaves [17]. *In Salmonella enterica*, mutants of FliGHI and CheY, deficient in motility and chemotaxis, respectively, resulted in poor stomatal penetration into lettuce leaves through their open stomata [18].

In this study, we investigated the factors responsible for the spatial colonization of PPFM in the phyllosphere. We studied the behavior of *Methylobacterium* sp. strain OR01 during the growth of red perilla, and followed its entire journey from seeds to the whole plant surface, and subsequently to the next-generation seeds. Fluorescent and electron microscopical observations elucidated the bacterial distribution and dynamic behavior in the phyllosphere. With the analyses of mutants impaired in FliC (the flagellin protein) and MptA of strain OR01, we demonstrate the importance of motility and methylotaxis in phyllosphere colonization of strain OR01.

## Materials and methods

### Bacterial strains and culture conditions

The bacterial strains used in this study are listed in Supplementary Table S1. *Methylobacterium* sp. strain OR01 and *M. aquaticum* strain 22A were grown at 28°C in the hypho minimal medium (hereafter referred to as hypho medium) (Supplementary Table S2) [19] containing one of the carbon sources described below and all of the B-group vitamins (Vitamin mix). Carbon sources were 0.5% or 0.05% (v/v) methanol, or 0.5% or 0.05% (w/v) disodium succinate. The antibiotic kanamycin (Km, 20 μg/mL) was added as needed. *Escherichia coli* HST08 premium competent cells (TaKaRa) were used for gene cloning and fluorescent protein expression. *E. coli* was grown at 37°C on LB medium (1% Bacto tryptone, 0.5% Bacto yeast extract and 0.5% NaCl) in the presence of ampicillin (50 μg/mL) or kanamycin (20 μg/mL). Growth was monitored by measuring the optical density at 600 nm (OD_600_).

### Construction of plasmids

The plasmids used in this study are listed in Supplementary Table S3, and the oligonucleotide primers are listed in Supplementary Table S4. Details of the plasmid construction and transformation of DNA to bacterial strains are described in Supplementary Information.

### Cultivation of red perilla

Red perilla seeds were surface-sterilized and grown under aseptic conditions using the following two cultivation set-ups. In one, seeds were sown on vermiculite in 20 cm-high plastic cups (PhytoTechnology Laboratories, LLC, C221) sealed with ventilated seals (Excel Scientific, Inc., B-100). 50 mL of HYPONex (HYPONex JAPAN Corp., Ltd.) diluted 2000 times from the original concentration was applied once every two weeks for 60 g of vermiculite per plastic cup. In the other, seeds were sown on Hoagland agar in 4 cm-high plastic culture dishes (SPL Life Sciences, Plant culture dish 100^*^40) prepared as described previously [8]. Vermiculite, Hoagland agar and plastic cups/dishes were autoclaved before use. The plants were placed in the NK Biotron LH-220 (Nippon Medical and Chemical Instruments, Osaka, Japan). The system was operated at 25°C, 65% humidity under a 15-h light 9-h dark cycle. Plants cultivated on agar media developed in the same way as plants grown in plastic cups containing vermiculite for the first 2 weeks. Thereafter, a dwarf phenotype was observed, but they appeared healthy otherwise.

### Measurement of cell populations of PPFMs on red perilla

Cells of strain OR01 and strain 22A were grown on the hypho medium containing 0.5% methanol and vitamin mix at 28°C for 2 days. After collecting, cells were washed with sterilized water and suspended in sterilized water to obtain a suspension with an OD_600_ of 0.1. Red perilla seeds were treated with 70% ethanol for 1 minute and with 1% (v/v) antiformin (containing 0.3% v/v Tween 20) for 5 minutes, followed by washing with sterilized water 5 times. They were soaked in 1 mL of the single or mixed (wild-type strain and each mutant strain) cell suspension for 3 hours with gentle shaking at 5 rpm using a Rotator RT-5 (Taitec, Saitama, Japan) at 28°C. The seeds incubated with *Methylobacterium* sp. OR01 were sown onto Hoagland agar in a plant culture dish (100 × 40 mm) or vermiculite for growth in the chamber. For the collection of PPFMs from red perilla, the whole leaves were cut and placed in a 1.5 mL tube, weighed, and then, 100 μL of PBS was added per 10 mg of leaf and vortexed for 15 minutes. 20 μL of this collection sample was mixed with 20 μL of BD™ Liquid counting Beads (BD Biosciences) and 200 μL of PBS and the total volume was used for flow cytometry (FCM) analysis to count the cell population.

### Analyses of the presence of bacterial cells in the stomata

Red perilla leaves were placed statically overnight on a bacterial solution of either strain OR01-GFP, strain Δ*mtpA*-GFP or strain Δ*fliC-*triple-GFP with an OD_600_ of 0.1. Microscopic analysis was performed with FV3000. Subsequently, ImageJ was used to measure the area of the open stomata from DIC images and the GFP fluorescent area of those open stomata from GFP images. Measured values were used for quantification of the ratio of (area of the open stomata with GFP fluorescence) / (total area of open stomata) (%). Similarly, the total number of the open stomata from DIC images and the number of those open stomata with GFP fluorescence from GFP images were measured. Measured values were used for quantification of the ratio of (number of open stomata with GFP fluorescence) / (number of all open stomata) (%).

### Capillary chemotaxis assay

Chemotaxis was evaluated using capillaries and a glass slide with a hole (Kenis, HS-1), as previously performed with some modifications [20]. Hypho medium supplemented with a carbon source at a concentration of 0.05%, as necessary, vitamin mix and appropriate antibiotics were sucked into a capillary, and then one end of the capillary was closed with nail polish. Subsequently, 200 μL of bacterial suspension of OD_600_ at 0.01 was applied to the hole portion of the glass slide with a hole and the capillary containing a carbon source was inserted into the hole. Then the hole was covered by a cover glass and this experimental set-up was incubated at 28°C for 3 hours. After culturing, the cells in the capillary were observed with an SZX16 fluorescence stereo microscope (Olympus). Black and white colors of the original image were then inverted. The number of cells in the capillary was examined by FCM.

### Flow cytometry

Flow cytometry was performed using the FACSAria™ III Cell Sorter (Becton Dickinson). Details are described in Supplementary Information.

### Microscopy

Microscopic analyses were conducted using the following microscopes; Fluorescence stereo microscope SZX16 (Olympus), IX73 inverted microscope (Olympus), confocal microscopes Zeiss LSM510 META/Axiovert 200 (Olympus), FV3000 (Olympus) and FV4000 (Olympus). Correlative light and electron microscopy (CLEM) analyses were performed using BX51 (Olympus) and electron microscope (Hitachi High-Tech SU8220).

Details of these microscopic analyses are described in Supplementary Information.

### Leaf-print assay

One month after aseptic growth, aerial parts of the plant were harvested. The harvested leaves of red perilla were cut and placed directly on the hypho medium agar plate containing 0.5% methanol or 0.5% succinate with the addition of kanamycin (20 μg/mL), as needed. Then, the leaf was gently pressed to the medium using a spreading stick and incubated at 28°C for 3–4 days. After the culture, microbial distribution was observed by the SZX16 fluorescence stereo microscope (Olympus). The leaf-print assay was used to qualitatively assess the distribution and colonization of bacteria on the leaf surface.

## Results

### Fluorescent analysis revealed the transmission of PPFM through plant tissue surfaces to seeds for the next generation

**Figure 1:**
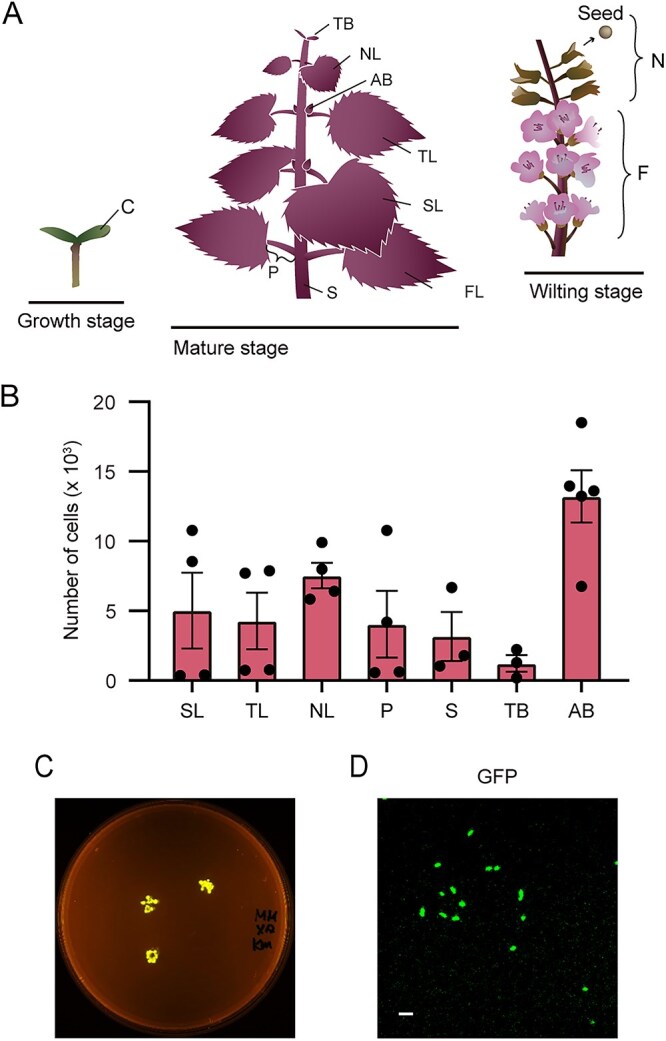
Colonization of *Methylobacterium* sp. strain OR01 from seeds to whole plant of red perilla through plant surface. (A) Schematic images of red perilla during plant development, i.e., growth, mature and wilting stages. C, cotyledon; TB, terminal bud; AB, axillary bud; P, petiole; S, stem; FL, first true leaf; SL, second true leaf; TL, third true leaf; NL, newest leaf; F, flower and N, next-generation seeds. (B) Quantitation of cell populations of strain OR01-mCherry on red perilla by FCM. Values are indicated as the number of cells per mg of plant sample and are shown as mean ± standard error of the mean (s.e.m.) of analyses with the harvested leaf samples. Leaf and petiole samples were prepared as follows: Four leaves were collected from two distinct plants, two leaves from each, and cut into leaf and petiole. As for terminal bud and stem, three samples were collected from three distinct plants, one sample from each. A total of five samples were collected from two plants for axillary bud samples. (C) The presence of strain OR01-GFP on next-generation seeds. The fruit-like structures enclosing the perilla seeds were harvested from red perilla flowers and those structures were directly placed on hypho medium agar plates containing 0.5% methanol for observation by FAS-Digi imaging system (NIPPON genetics) after 3 days. Some 20–30 fruit-like structures were harvested from one flower of red perilla. (D) Microscopic images of the strain OR01-GFP collected from next-generation seeds. Cells of strain OR01-GFP were isolated by toothpicks directly from the seeds used in (C) under FAS-Digi imaging system and placed onto a slide glass for observation. GFP-fluorescence image was demonstrated. Bar, 2 μm.

We constructed the *Methylobacterium* sp. strain OR01 expressing GFP (strain OR01-GFP) and confirmed that expression of the fluorescent protein did not affect the growth of strain OR01 (Supplementary Fig. S1). To follow the bacterial behavior, strain OR01-GFP was inoculated onto seeds of red perilla. Three to four months after inoculation, aerial parts of the plant (the second leaf; SL, the third leaf; TL, newest leaf; NL, petiole; P, stem; S, terminal bud; TB, axillary bud; AB, and flower; F, as well as the next-generation seeds; N, picked up from the seed bag of the flower) were harvested at various stages (Fig. 1A). The collected samples were rinsed in sterile water and spread onto a hypho medium agar plate supplemented with 0.5% methanol as a single carbon source. GFP-fluorescent colonies appeared on all the tested plates, confirming the presence of strain OR01-GFP on the surface of the entire red perilla plant (Supplementary Fig. S2). We constructed the strain OR01 expressing mCherry (strain OR01-mCherry) (Supplementary Fig. S1) and quantified the cell number using flow cytometry (FCM) analysis to examine the localization of the bacteria. Similar to colony formation analysis, aerial parts of the perilla plant were harvested three to four months after seed-inoculation of strain OR01-mCherry. FCM analysis revealed that about 13 000 cells were present per mg of the axillary bud, while approximately 2000 cells were present on the petiole, terminal bud and stem (Fig. 1B). Among the leaf samples, the largest number of cells was detected with new leaf samples. These results indicated that a higher number of bacteria colonize younger plant tissues such as axillary bud and the newest leaves. We also harvested fruit-like structures from flowers and placed them onto a hypho medium agar plate containing 0.5% methanol for direct observation by a transilluminator imaging system (Fig. 1C). In addition, cells of strain OR01 were isolated from the seeds placed on the agar plate for microscopic observation (Fig. 1D). We detected GFP fluorescence from the plant and bacterial samples, demonstrating that strain OR01-GFP traveled throughout the surface of plant tissues, and colonized the next-generation seeds.

### P‌PFM preferentially resides around the periphery, at the base of the trichome, along the vein and around the stomata

**Figure 2:**
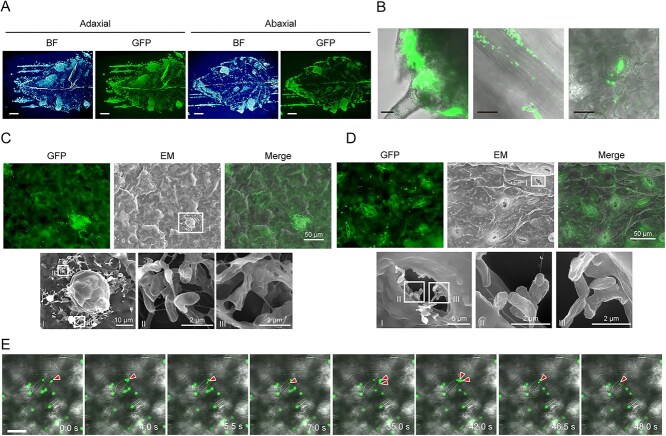
Distribution of *Methylobacterium* sp. strain OR01 on red perilla leaves. (A) Leaf-print assay of strain OR01-GFP on red perilla leaves at the adaxial side (left) and abaxial side (right). Bright-field (BF) image (left) and GFP-fluorescence image (right) are presented. Bar, 2 mm. (B) Confocal fluorescence microscopic images showing strain OR01-GFP distributed at the base of the trichome (left), along the vein (central) and stomata (right). Bar, 20 μm. (C-D) CLEM images of strain OR01-GFP around the trichome (C) and stomata (D). GFP fluorescence images (upper left) and EM images (upper central) were used for merged CLEM images (upper right). The EM images highlighted with a square (I) were magnified (lower left). The areas marked as square (II) and (III) were further magnified (II) lower central and (III) lower right, respectively. Bars show the indicated lengths. (E) Time-lapse images of strain OR01-GFP entering the stomatal cavity. The video was taken by a confocal microscope and recorded for 77 seconds. Eight images extracted at the indicated time points are shown (see also Supplementary Movie S2). Red arrows indicate cells entering into the stomatal cavity from the surface of the leaf. Bar, 20 μm.

To investigate the bacterial distribution on plant leaves, a leaf-print assay was performed with the first true leaves after one month of bacterial inoculation on seeds. We found that strain OR01-GFP was distributed throughout both adaxial and abaxial sides of the leaves with a preference to sites around the periphery (Fig. 2A). A confocal microscopic analysis found that bacterial colonies were present particularly at the base of the trichomes (Fig. 2B left image), along the vein (central image) and around the stomata (right image). Interestingly, GFP fluorescence was detected not only around but also inside the stomata. The z-stack images found that a substantial number of strain OR01-GFP was present in the sub-stomatal cavity of cotyledon (Supplementary Fig. S3A and Supplementary Movie S1).

To further analyze strain OR01-GFP on the leaf surface, CLEM was performed. We found that numerous clumps were present throughout the leaf surface (Supplementary Fig. S3B). Enlarged images revealed that strain OR01-GFP cells were present around the trichome in biofilm-like structures that were about 20 μm x 20 μm long vertically and horizontally (Fig. 2C), in a similar manner to a previous study [21]. Furthermore, CLEM captured the presence of strain OR01-GFP around the stomata (Supplementary Fig. S3C). Under higher magnification, strain OR01-GFP was detected inside the stomata (Fig. 2D), as demonstrated in Fig. 2B and Supplementary Fig. S3A with the confocal microscope. To investigate the bacterial dynamics around the stomata, 2 hours after soaking the leaf in the bacterial suspension, we monitored the strain OR01-GFP that existed around the stomata over time. Interestingly, the videography captured the moments of strain OR01-GFP entering the stomata (Fig. 2E and Supplementary Movie S2). Within the recorded 77 seconds, four cells of strain OR01-GFP moved into the stomata (Supplementary Movie S2). We constructed the *E. coli* expressing GFP with no motility (HST08-GFP) and observed their dynamics on the leaf surface. However, none of the HST08-GFP cells on the leaf surface moved into the stomatal cavity (Supplementary Movie S3), suggesting that bacterial motility contributes to their distribution in the phyllosphere.

### FliC (flagellin)-dependent motility and bacterial distribution in the phyllosphere

Our observation of the cell entry into the stomata prompted us to speculate that bacterial distribution in the phyllosphere is affected by flagellin-dependent motility. We constructed a gene-deletion strain OR01 lacking all of the flagellin proteins, i.e., *fliC1*, *fliC2* and *fliC3*, that expressed GFP (strain Δ*fliC-*triple-GFP). Deletion of these three genes resulted in complete loss of flagellum (Supplementary Figs. S4A-B) and loss of motility (Supplementary Figs. S4C-D and Supplementary Movies S4-S5).

**Figure 3:**
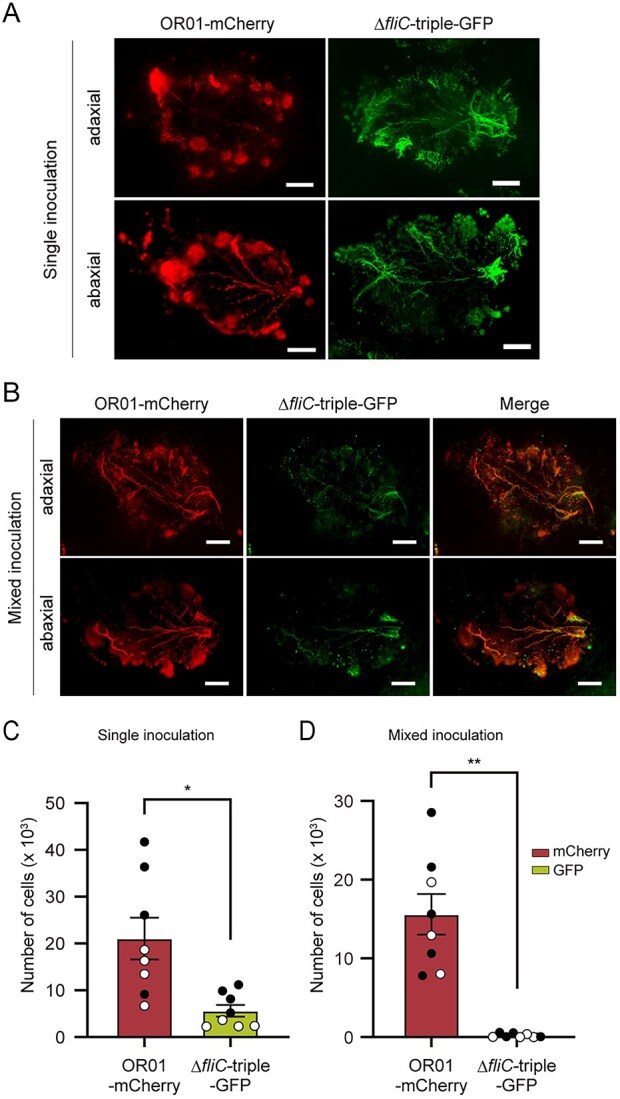
Colonization and distribution of FliC (flagellin)-deleted mutant of strain OR01 in the phyllosphere of red perilla. (A-B) Visualization of colonization and distribution of strain OR01-mCherry and strain Δ*fliC*-triple-GFP on red perilla by leaf-print assay. Strain OR01-mCherry and strain Δ*fliC*-triple-GFP were inoculated in a single inoculum (A) or in a mixed inoculum (B). Bar, 2 mm. (C-D) FCM-based quantification of cell populations of strain OR01-mCherry and strain Δ*fliC*-triple-GFP on red perilla. Strain OR01-mCherry and strain Δ*fliC*-triple-GFP were inoculated in a single inoculum (C) or in a mixed inoculum (D). Values are indicated as the number of cells per mg of plant sample and are shown as mean ± s.e.m. of analyses with eight leaves. Of the eight leaves collected from two distinct plants, four were first leaves (black circles) and the remaining four were second leaves (white circles). Asterisks indicate the level of statistical significance: ^**^P < .01, ^*^P < .05.

We inoculated strain Δ*fliC-*triple-GFP and strain OR01-mCherry on red perilla seeds and harvested the first and second true leaves one month after cultivation. Leaf-print assay revealed that mCherry fluorescence of strain OR01-mCherry and GFP fluorescence of strain Δ*fliC*-triple-GFP were detected under both single and mixed inoculation conditions (Figs. 3A-B). FCM-based quantitative analysis counted approximately 20 000 cells of strain OR01-mCherry and 5500 cells of strain Δ*fliC-*triple-GFP per mg of perilla leaf under single inoculation conditions (Fig. 3C). Under mixed inoculation conditions, the difference between these strains became more pronounced in that the cell number of strain OR01-mCherry remained around 15 000, whereas that of strain Δ*fliC-*triple-GFP was reduced to less than 300 (Fig. 3D). These results suggested that bacterial motility promoted phyllosphere colonization of strain OR01 during transmission from seeds to the phyllosphere.

### MtpA-dependent methylotaxis accelerates stomatal entry and colonization of PPFM to the leaf surface

Bacterial chemotaxis is driven by a signal transduction mechanism involving MCPs and Che proteins and the directional movement controlled by the flagellin protein FliC. To further investigate the involvement of chemotaxis in phyllosphere colonization, we focused on methylotaxis. We disrupted *mtpA* (homologous to methylotaxis protein A found in *M. aquaticum* strain 22A) (MaMtpA, identity: 49%, similarity: 63%) in the genome of strain OR01 (Supplementary Figs. S5A-B), as the amino acid sequence showed the highest similarity to MaMtpA among 34 MCPs identified in the draft genome sequence of strain OR01. We found that MtpA in strain OR01 contains an MCP signaling domain, similarly to MaMtpA in strain 22A, and that neither MtpA in strain OR01 nor MaMtpA in strain 22A contained a transmembrane domain. To investigate the role of MtpA in the chemotaxis of strain OR01, we performed a capillary chemotaxis assay using strain OR01-mCherry and the strain Δ*mtpA* expressing GFP (strain Δ*mtpA*-GFP). Stereo microscopy revealed that many cells of strain OR01-mCherry, detected as black specks between the capillaries in the image, were present when methanol was supplemented, whereas few cells of strain Δ*mtpA*-GFP were detected in the capillaries. In contrast, substantial numbers of cells of both strain OR01-mCherry and strain Δ*mtpA*-GFP were present in the capillaries when succinate was supplemented (Supplementary Fig. S6A). FCM analyses further revealed that deletion of *mtpA* led to a significant reduction in methylotaxis of strain OR01 without disrupting chemotaxis to succinate (Supplementary Fig. S6B), indicating that MtpA is a major chemoreceptor for methanol in strain OR01. Notably, methylotaxis was lost in strain 22A when all three genes responsible for methanol-sensing chemoreceptors were disrupted [16], whereas in strain OR01 the disruption of *mtpA* resulted in the loss in methylotaxis.

**Figure 4:**
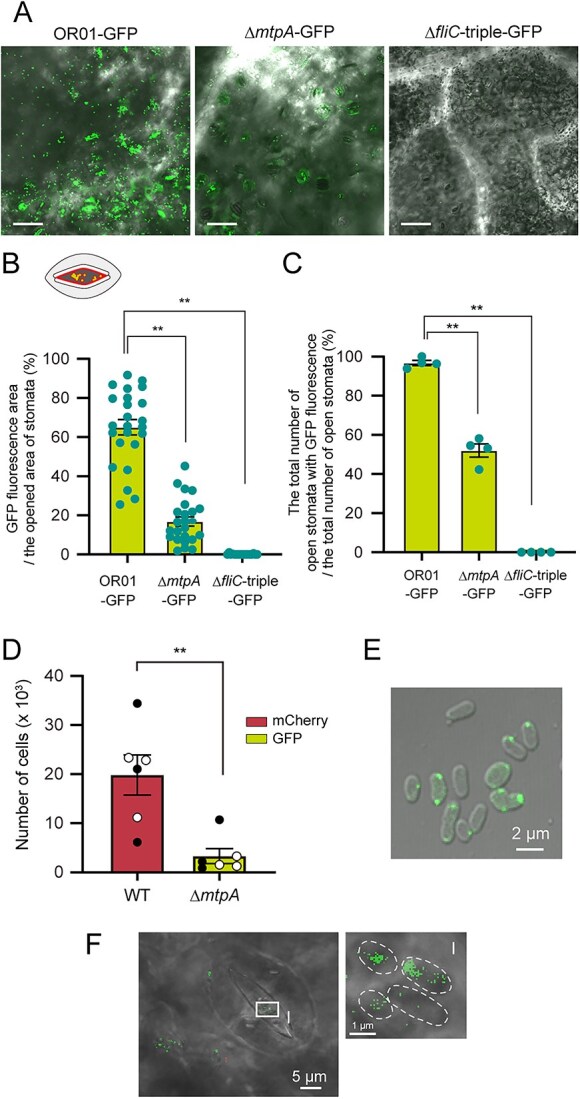
MtpA-dependent methylotaxis affected the distribution and colonization of strain OR01 in the phyllosphere of red perilla. (A-C) The strain OR01-GFP, strain Δ*mtpA*-GFP, and strain Δ*fliC*-triple-GFP present in a single stomata. (A) Representative confocal microscopic images of strain OR01-GFP, strain Δ*mtpA*-GFP, and strain Δ*fliC*-triple-GFP on red perilla leaves. Marged images of the DIC and GFP images are shown. Bar, 50 μm. (B) Quantification of the ratio of (area of the open stomata with GFP fluorescence) / (total area of open stomata) (%). Values are indicated as the number of cells per mg of plant sample and are shown as mean ± s.e.m. of the analysis of 18 stomata from three distinct leaves in different plants. These leaves were harvested 70 days after seed planting with the inoculation of bacterial cells. Asterisks indicate the level of statistical significance between strain OR01-GFP and strain Δ*mtpA*-GFP, and strain OR01-mCherry and strain Δ*fliC*-triple-GFP: ^*^P < .05, ^**^P < .01. (C) Quantification of the ratio of (number of open stomata with GFP fluorescence) / (number of all open stomata) (%). Values are indicated as the number of cells per mg of plant sample and are shown as the mean ± s.e.m. of the analysis of 60 stomata from three distinct leaves. Asterisks indicate the level of statistical significance between strain OR01-GFP and strain Δ*mtpA*-GFP, and strain OR01-GFP and strain Δ*fliC*-triple-GFP: ^**^P < .01. (D) FCM-based quantification of cell populations of strain OR01-mCherry and strain Δ*mtpA*-GFP on red perilla. Values are indicated as the number of cells per mg of plant sample and are shown as mean ± s.e.m. of analyses with six leaves. Of the six leaves collected from two distinct plants, three were second leaves (black circle) and the remaining three were third leaves (white circle). Asterisks indicate the level of statistical significance between strain OR01-GFP and strain Δ*mtpA*-GFP: ^**^p < 0.01. (E) Confocal microscopic images of strain Δ*mtpA*-GFP-MtpA. Cells were cultured on hypho medium containing 0.5% methanol as a carbon source. Marged image of the DIC and GFP images is shown. Bar, 2 μm. (F) Confocal microscopic images of strain Δ*mtpA*-GFP-MtpA on the red perilla leaf surface. The original DIC- and GFP-merged image is shown. The highlighted square (I of panel F) is magnified, and shown in a DIC- and GFP-merged image. Bars show the indicated lengths. Cell shapes are highlighted with dotted lines in the merged image.

To investigate the cell entry to the stomata, leaves were placed statically overnight on a bacterial solution of either strain OR01-GFP, strain Δ*mtpA*-GFP or strain Δ*fliC-*triple-GFP. Microscopic analysis demonstrated that the cell number of strain Δ*mtpA*-GFP present in the stomata was lesser than that of strain OR01-GFP (Fig. 4A). In addition, strain Δ*fliC-*triple-GFP, which revealed that the lack of FliC proteins, resulted in further reduction in the number of cells detected compared to that in strain Δ*mtpA*-GFP (Fig. 4A). These results indicated that the motility driven by MtpA and FliC is critical for bacterial entry into the stomata and eventually for colonization on the red perilla leaf surface. Quantitative analysis revealed that 65% of the total area of open stomata was covered by GFP fluorescence of strain OR01-GFP, whereas only 17% and 0.1% were covered by GFP fluorescence of strain Δ*mtpA*-GFP and strain Δ*fliC-*triple-GFP, respectively (Fig. 4B). In addition, at least one cell of strain OR01-GFP was observed in more than 95% of the stomata, whereas cells of strain Δ*mtpA*-GFP were present in approximately 52% of the stomata and those of strain Δ*fliC-*triple-GFP were not detected (Fig. 4C), demonstrating that MtpA is the major MCP amongst others that are responsible for the entry of strain OR01 into the stomatal cavity.

Subsequently, we investigated the physiological significance of MtpA-dependent methylotaxis in bacterial colonization in the phyllosphere of red perilla. After sterilization, red perilla seeds were inoculated with either strain OR01-mCherry or strain Δ*mtpA*-GFP. Two months after plant growth under single inoculation conditions with bacterial cells, we performed quantitative analysis by FCM and revealed that an average of 20 000 cells per mg of the plant sample was detected from the leaf inoculated with strain OR01-mCherry, whereas only about 4000 cells of strain Δ*mtpA*-GFP were counted from the collected leaf sample (Fig. 4D), suggesting that methylotaxis plays a critical role in the colonization of strain OR01 in the phyllosphere. Although the *mtpA* deletion led to a significant reduction in the bacterial entry into the stomatal cavity and colonization on the leaf surface, its impact was not as large as the complete loss of flagellin (Figs. 4A-C).

To investigate the intracellular localization of MtpA in strain OR01, we constructed the Δ*mtpA* strain expressing GFP-MtpA fusion protein (strain Δ*mtpA*-GFP-MtpA). The functionality of the fusion protein was confirmed by complementation experiment of methylotaxis, using strain OR01, strain Δ*mtpA* and strain Δ*mtpA*-GFP-MtpA (Supplementary Fig. S6C). During cultivation on methanol, GFP-MtpA signal was found to form foci and localize mainly to the poles of cells (Fig. 4E), suggesting that MtpA in strain OR01 forms the chemotaxis sensory array at the cell pole together with Che proteins, in a manner similar to that previously demonstrated in other MCPs [22]. Notably, MaMtpA in strain 22A localizes to the cytosol [16], while MtpA in strain OR01 localizes at the cell pole, although both methanol-sensing chemoreceptors do not contain a transmembrane domain. Subsequently, we inoculated strain Δ*mtpA*-GFP-MtpA onto the leaf surface of red perilla and found that GFP-MtpA localized to specific sites in the cytosol (Fig. 4F). These results suggested that MtpA is responsible for methylotaxis in the phyllosphere.

### The predominant colonization of strain OR01 on red perilla is attributed to its high methylotaxis activity

**Figure 5:**
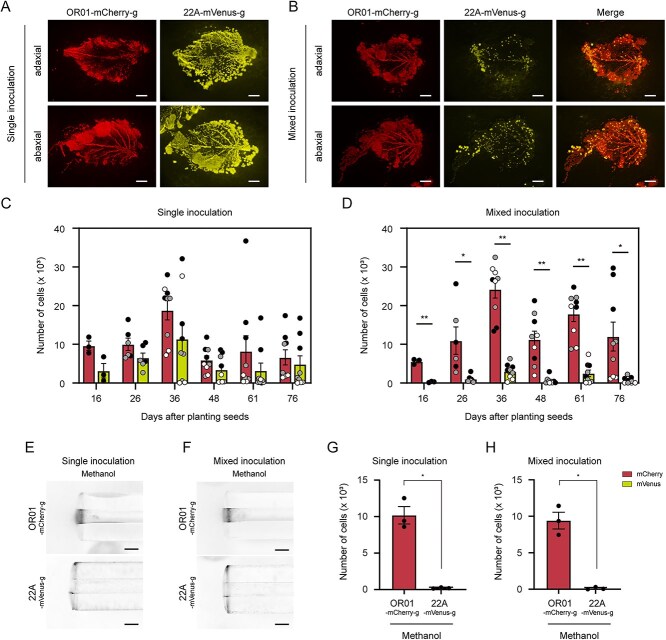
Competitive colonization analysis between strain OR01 and strain 22A on red perilla. (A-B) Leaf-print assay of strain OR01-mCherry-g and strain 22A-mVenus-g on red perilla leaves. These strains were inoculated in a single inoculum (A) or a mixed inoculum (B). Bar, 2 mm. (C-D) FCM-based quantification of the cell populations of strain OR01-mCherry-g and strain 22A-mVenu-g on red perilla leaves. The first to third true leaves were harvested 16, 26, 36, 48, 61, and 78 days after seed planting with the inoculation of bacterial cells. Strain OR01-mCherry-g and strain 22A-mVenus-g were inoculated in a single inoculum (C) or in a mixed inoculum (D). Values are indicated as the number of cells per mg of plant sample and are shown as mean ± s.e.m. of analyses with at the harvested leaf samples. A total of three first leaves were collected from two distinct plants on Day 16, three first leaves and three second leaves from two distinct plants on Day 26, and nine first leaves, three second leaves, and three third leaves from two distinct plants on Days 36, 48, 61, and 76. The number of cell populations obtained from the first, second, and third leaves are indicated by black, gray and white circles, respectively. Asterisks indicate the level of statistical significance between strain OR01-mCherry-g and strain 22A-mVenu-g on each indicated date: ^**^P < .01, ^*^P < .05. (E-H) Capillary assay for methylotaxis of strain OR01-mCherry-g and strain 22A-mVenus-g. (E-F) The strains were placed close to the capillaries that contained a hypho medium supplemented with 0.05% methanol. The strains were inoculated in a single inoculum (E) or in a mixed inoculum (F). Images were taken by a stereo microscope. Bar, 200 μm. (G-H) FCM-based quantification of cell populations of strain OR01-mCherry-g and strain 22A-mVenus-g in capillaries. The strains were inoculated in a single inoculum (G) or in a mixed inoculum (H). Values are indicated as the number of cells per capillary and are shown as mean ± s.e.m. of analyses with three distinct capillaries. Asterisks indicate the level of statistical significance between strain OR01-mCherry-g and strain 22A-mVenus-g: ^*^P < .05.

Strain OR01 is predominant in the phyllosphere of red perilla [8]. Next, we set out to analyze the reason for this predominance by performing a competitive colonization analysis of mCherry-expressing strain OR01 (strain OR01-mCherry-g) with mVenus-expressing *M. aquaticum* strain 22A (strain 22A-mVenus-g). Similarly to strain OR01, we confirmed that expression of the fluorescent protein did not affect the cell growth of strain 22A (Supplementary Fig. S7). After one month of the bacterial inoculation on seeds, we found that strain OR01-mCherry-g and strain 22A-mVenus-g were distributed throughout the leaf surface both on the adaxial and abaxial sides under a single inoculation condition (Fig. 5A). However, inoculation conditions in a mixed inoculum led to a substantial decrease in the fluorescence of mVenus of strain 22A-mVenus-g cells in comparison with that of strain OR01-mCherry-g on both sides of the leaf surface (Fig. 5B). mCherry fluorescence in OR01-mCherry-g was distributed throughout the leaf surface, while mVenus in 22A-mVenus-g was scattered as dots and did not spread uniformly. To quantify the number of cells, the first, second, and third true leaves were collected regularly for FCM analysis. Under inoculation conditions in a single inoculum, ca. 5000 to 20 000 cells of strain OR01-mCherry-g and ca. 2500 to 12 000 cells of strain 22A-mVenus-g were detected per mg perilla leaf, and there was no statistical difference between these two strains (Fig. 5C). With mixed inoculum of both strains, we found a significant difference in that the cell number of strain OR01-mCherry-g was ca. 5000 to 25 000, whereas that of strain 22A-mVenus-g was 300 to 3000 (Fig. 5D). These results indicated that strain OR01 is more competitive than strain 22A in colonization on red perilla.

Finally, we explored the characteristics that allowed strain OR01 to be competitive on red perilla by a capillary chemotaxis assay. Analysis by stereo microscopy revealed that methylotaxis activity of strain OR01-mCherry-g was much higher than that of strain 22A-mVenus-g both under single and mixed inoculation conditions (Figs. 5E-F and Supplementary Figs. S8A-B). FCM analysis quantified that the cell number of strain OR01-mCherry-g was approximately 10 000, whereas that of strain 22A-mVenus-g was 200 with a single inoculum (Fig. 5G). With a mixed inoculum, the cell number of strain OR01-mCherry-g was 10 000, whereas that of strain 22A-mVenus-g was reduced to 100, which was consistent with the analysis by stereo microscopy (Fig. 5H). To explore the sensitivity of strain OR01 to methanol further, we performed a capillary chemotaxis assay supplemented with different concentrations of methanol. We found that strain OR01 migrated into a capillary with the cell numbers showing the correlation with the methanol concentration as low as 0.000012%, demonstrating the remarkable sensitivity of strain OR01 to methanol (Supplementary Fig. S9).

## Discussion

**Figure 6:**
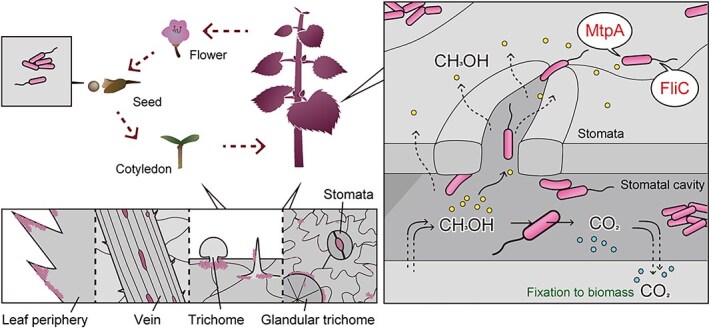
MtpA-dependent methylotaxis and FliC-driven motility enhance the colonization of *Methylobacterium* sp. strain OR01 in the phyllosphere of red perilla. Strain OR01 travels throughout the plant surface in the phyllosphere and moves to seeds for the next generation concomitant with plant growth. Strain OR01 preferentially colonizes the leaf periphery, veins, trichomes, and stomata. Methanol generated from pectin in the plant cell wall functions as a major volatile messenger to attract strain OR01 into the stomatal cavity aided by MtpA-driven methylotaxis and FliC-dependent motility. Within the stomatal cavity, strain OR01 oxidizes methanol to CO_2_, resulting in an increase in CO_2_ concentration used for photosynthesis by the host plant.

This study demonstrates that methanol-sensing MtpA, which drives methylotaxis, determines the spatial colonization of *Methylobacterium* sp. strain OR01 in the phyllosphere of its natural host, the red perilla plant. During its transmission, strain OR01 was present on the entire leaf surface with a preference for sites around the periphery, vein, trichome and stomata of the leaf surface. Strain OR01 entered the sub-stomatal cavity attracted by methanol and this behavior was attributed to MtpA-driven methylotaxis and FliC-dependent motility. Furthermore, their high methylotaxis activity contributed to the competitiveness of strain OR01 on red perilla (Fig. 6).

Of particular interest is the MtpA-dependent entry of strain OR01 into the stomata (Fig. 2E and Supplementary Movie S2). Previously, we developed a methanol cell sensor and directly determined the local methanol concentration on the leaf surface of *Arabidopsis thaliana* to be 0–0.21% (0–64 mM) with a periodic methanol oscillation, i.e. high in the dark and low in the light period [13], which might be directly linked to the hydrolyzation of pectin in the plant cell wall. We confirmed the high methanol-sensing ability of strain OR01 using a capillary assay, which showed that strain OR01 entered the capillary supplemented with 0.000012% methanol (Supplementary Fig. S9). The methanol sensing machinery comprising Wsc1 and Wsc3 in the yeast *Komagataella phaffii* senses methanol concentration in the range between 0.005 and 1%. In comparing the bacterial and yeast methanol-sensing machineries, MtpA in strain OR01 is 500-fold more sensitive than the yeast methanol sensor Wsc, suggesting that methanol not only serves as a carbon source for strain OR01, but also functions as a volatile messenger for communication with the host plant.

One possible benefit for the host plant to attract PPFMs to the stomatal cavity is the *in-situ* oxidation of methanol to CO_2_ that can be directly fixed by plants through photosynthesis (Fig. 6). A previous study with ^13^C-methanol labeling and liquid chromatography-mass spectrometry analyses suggested that methanol present on the leaf surface was primarily oxidized to CO_2_ for energy generation in PPFM, although a portion of the methanol entered into the methanol assimilation pathway [23]. Another benefit of bacterial presence in the stomatal cavity for plants may be the increased protection from pathogen invasion. *P. syringae*, a widely studied plant pathogen, is known to enter the stomatal cavity during infection [24–28]. Therefore, attracted by methanol, PPFMs may move to the stomatal cavity and occupy the colonization place before pathogens. Other than the stomatal cavity, invasion of pathogens to the host cells yielded methanol by secreted pectinases [29], which might attract PPFM to the invasion site.

Previously, we reported that many PPFMs isolated from plant samples require B vitamins for growth on minimal media, e.g., pantothenate (vitamin B_5_) [19]. It may be advantageous for PPFMs to colonize sites like glandular trichome to acquire nutrients other than methanol that are necessary for bacterial growth [30].

Deletion of the *mtpA* gene responsible for methylotaxis and *fliC* genes required for motility led to a significant decrease in the number of cells entering the stomata and colonizing red perilla leaves (Figs. 4A-C). The role of chemotaxis and/or motility in bacterial colonization has been studied extensively with rhizosphere bacteria, and previous studies have revealed their significance in competitive root surface colonization and establishment of a successful symbiosis [31–34]. In addition to colonization and symbiosis, transmission is also reported to be supported by bacterial movement [35]. The physiological role of chemotaxis has also been investigated using pathogenic bacteria. A previous report has identified AreA and AreB, the genes responsible for chemotaxis in *P. syringae*, as contributing to the pathogenic invasion of the host plant [36]. Another study has found that chemotaxis to nitrate and nitrite contributes to the colonization of the pathogen *Dickeya dadantii* on its host plant [37]. However, insights from these studies have been limited to the rhizosphere, in particular to nitrogen fixation bacteria and the loci for pathogenic invasion. Our study has shed light on bacterial chemotaxis and motility of phyllosphere microorganisms by demonstrating their crucial role in colonizing the host plant through its entire life cycle, rather than being confined to a specific part or developmental stage of the plant. To our knowledge, this is also the first report to show methanol as a compound that attracts phyllosphere bacteria to enter stomatal pores.

## Supplementary Material

ISME-Com_Supplementary_20250524_ycaf092

Supplementary_Movie_1_ycaf092

Supplementary_Movie_2_ycaf092

Supplementary_Movie_3_ycaf092

Supplementary_Movie_4_ycaf092

Supplementary_Movie_5_ycaf092

## Data Availability

The nucleotide sequences of *fliC1*, *fliC2*, *fliC3*, *mtpA*, and *mxaF* were deposited in the DDBJ/EMBL/GenBank under accession numbers LC833873, LC833874, LC833875, LC833876 and LC833877, respectively. All data generated or analysed during this study are included in this published article and its supplementary information files.
