# Supplementary material for: Methanol chemoreceptor MtpA- and flagellin protein FliC-dependent methylotaxis contributes to the spatial colonization of PPFM in the phyllosphere
Source: ISME Commun. 2025 May 29;5(1):ycaf092. doi: 10.1093/ismeco/ycaf092 (PMC12202990; doi:10.1093/ismeco/ycaf092)
Supplement: ISME-Com_Supplementary_20250524_ycaf092 [file isme-com_supplementary_20250524_ycaf092.pdf]

Supplementary Information

Methanol chemoreceptor MtpA- and flagellin protein FliC-dependent methylotaxis contributes to the spatial colonization of PPFM in the phyllosphere

Shiori Katayama, Kosuke Shiraishi, Kanae Kaji, Kazuya Kawabata, Naoki Tamura, Akio Tani, Hiroya Yurimoto* and Yasuyoshi Sakai

*Hiroya Yurimoto E-mail: yurimoto.hiroya.5m@kyoto-u.ac.jp

Extended Materials and Methods

Construction of plasmids

The plasmid vectors pBluescript II SK(+) (Stratagene Inc), pDCG-1[1], pMO149[2], pAT02-V[3], pCM80-Km[4], pCM1682[5] and pK18mobsacB[1] were used in previous studies.

The vector pBS-P_{mxaF} was constructed as follows: The promoter region of the gene *mxaF* was PCR-amplified by using the genomic DNA of *Methylobacterium* sp. strain OR01 with a primer pair PmxaF-Fw-KpnI/PmxaF-Rv-HindIII. After digestion with *Kpn*I and *Hind*III, the DNA fragment was ligated with linearized pBluescript II SK(+) with the same set of restriction enzymes. Subsequently, the primer pairs GFP-Fw-HindIII/GFP-Rv-PstI and mCherry-Fw-HindIII/mCherry-Rv-BamHI were used to amplify the 0.7-kb *GFP* and *mCherry* genes with pDCG-1 and pMO149 as templates, respectively. The PCR products with *GFP* and *mCherry* sequences were ligated into the linearized pBS-PmxaF by restriction enzyme sets, *Hind*III-*Pst*I and *Hind*III-*Bam*HI, respectively, resulting in the pBS-PmxaF-GFP and pBS-PmxaF-mCherry vectors.

The pCM802-based vectors were constructed from a plasmid vector pCM80-Km given by Dr Toshiaki Fukui. The *mxaF* promoter region of *M. extorquens* strain AM1 was removed using a primer set pCM80km-Re1800/pCM80km-Fw2471 by inverse PCR with pCM80-Km as a template, followed by self-ligation, yielding pCM802-Km. The vectors pCM802-Km and P_{mxaF}-GFP were digested by *Kpn*I and *Pst*I and ligated to each other, resulting in the plasmid pCM802-GFP. Similarly, the vectors pCM802-Km and P_{mxaF}-mCherry were cut by *Kpn*I and *Sph*I, and these DNA fragments were ligated to obtain the plasmid pCM802-mCherry. The vector pCM802-GFP-mtpA was constructed as follows: The promoter and ORF regions of *mtpA* were PCR-amplified using primer pairs NB_PmtpA_Fw/NB_PmtpA+GFP_Rv and NB_PmtpA+GFP_Fw/NB_GFP+mtpA_Rv with the genomic DNA of strain OR01 as templates. The *GFP* coding sequence was amplified by PCR using a primer pair NB_GFP+mtpA_Fw/NB_mtpA_Rv with pCM802-GFP as a template. These two DNA fragments were cloned into the *Bam*HI and *Sac*I sites of pCM802-Km by NEbuilder cloning kit (New England Biolabs), resulting in the vector pCM802-GFP-mtpA. These pCM802-based vectors were used as vectors for expression in plasmids.

The plasmid vectors pCM1684 and pCM1685 for genome insertion into strains OR01 and 22A, respectively, were constructed as follows: pCM1684 was constructed by ligating

pCM1682-derived sequences with a strain OR01 chromosome region at 404241 locus where no gene was assigned, and further with P_{mxaF}-mCherry sequence amplified from pCM802-P_{mxaF}-mCherry. Similarly, pCM1685 was constructed by ligating pCM1682-derived sequences with a strain 22A chromosome region at 1718735 locus where no gene was assigned, and further with P_{mxaF}-Venus sequence amplified from pAT02-Venus. These ligation processes were achieved by an NEbuilder cloning kit, resulting in the vector pCM802-GFP-mtpA.

The plasmid vectors for precise in-frame deletion of *mtpA*, *fliC1*, *fliC2* and *fliC3* genes were constructed as follows: pK18mobsacB carrying a positive selection marker (Km^r gene) and a counter-selectable marker (suicide gene *sacB*) was used for constructing all the pK18-based plasmid vectors for gene disruption, as previously describe[3]. Subsequently, the disruption vector pK18 ΔfliC1 was constructed as follows: The *fliC1* and the homologous fragment flanking *fliC1* were PCR-amplified using a primer pair fliC1_up_Fw/ fliC1_down_Rv with the genomic DNA of strain OR01 as a template. The DNA fragment and pK18mobsacB, digested by restriction enzymes *Sal*I and *Sph*I, were ligated to each other, resulting in the plasmid pK18 fliC1. The *fliC1* ORF region was removed using a primer set Inv_fliC1_Fw1/ Inv_fliC1_Rv by inverse PCR with pK18 fliC1 as a template, followed by a self-ligation, yielding the vector pK18 ΔfliC1. The disruption vectors pK18 ΔfliC2 and pK18 ΔmtpA were constructed in a similar manner. The ORF, upstream and downstream region of *fliC2* and *mtpA* were PCR-amplified using primer pairs fliC2_up_Fw/ fliC2_down_Rv and mtpA_up_Fw/mtpA_down_Rv, respectively, with the genomic DNA of strain OR01 as a template. The DNA fragments and pK18mobsacB, digested by restriction enzymes *Sal*I and *Sph*I, were ligated to each other, resulting in the plasmid pK18 fliC2 and pK18 ΔmtpA. The disruption vector pK18 ΔfliC3 was constructed as follows: the homologous fragments flanking *fliC3* were amplified by PCR using primer pairs fliC3_up_Fw/ fliC3_up_Rv and fliC3_down_Fw/ fliC3_down_Rv with the genomic DNA of strain OR01 as a template. These fragments were cloned into the *Sal*I and *Sph*I sites of pK18mobsacB by an NEbuilder cloning kit.

Transformation of DNA to *Methylobacterium* sp. OR01 and *M. aquaticum* strain 22A

Cells were grown for 48 hours and 500 µL of them were centrifuged at 15,000 rpm for 1 minute. To prepare for competent cells of *Methylobacterium* sp. OR01, harvested cells were washed with sterilized water and then resuspended in 50 µL of sterilized water. For *M. aquaticum* strain 22A,

collected cells were washed with 10% glycerol and then resuspended in 50 μL of sterilized water containing 10% glycerol and 30% PEG. Competent cells were mixed with 1 μL of DNA and transferred to 1 mm gap cuvette (Bio-Rad). The DNA was introduced to the cells by electroporation at 1.8–2 kV, 25 μF, 200 Ω (GenePulser Xcell™, Bio-Rad). The entire electroporated suspension was inoculated into 500 μL Nutrient Broth (Difco™) medium and cultured for 3 hours at 28°C. These cells were plated on the hypho medium plates containing 0.5% succinate and vitamin mix and incubated at 28°C for 3–5 days. For obtaining gene knockout strains, the grown colonies were streaked on R2A agar medium containing 10% sucrose, 0.5% succinate and 1% vitamin mix. After culturing at 28°C for about 5 days, mutant strains were selected by colony PCR.

Flow cytometry

We used the FACSAria™ III Cell Sorter (Becton Dickinson). Fluorescent channel and light scatter were set at log gain. The forward scatter (FSC) was set at a photomultiplier tube (PMT) voltage of 2220 with a threshold of 200. The PMT voltages of side scatter (SSC) was set at 250 with a threshold of 200. Green fluorescent protein (GFP), mVenus and mCherry wereset at 445, 515 and 470, respectively. GFP and mVenus fluorescences were excited with a 488-nm laser, and the emission at 530/30 nm was detected. mCherry fluorescence was excited with a 561-nm laser, and the emission at 610/20 nm was detected. Cells were counted referring to 2000 quantitative beads as standards. To ensure that FCM sorts GFP-labelled, mVenus-labelled and mCherry-labelled cells properly, we set gates for these labelled cells by using the GFP-labelled, mVenus-labelled and mCherry-labelled cells cultivated in hypho medium supplemented with 0.05% methanol. FACSDiva 8 software (Becton Dickinson) was used for data acquisition.

Microscopes

Fluorescence stereo microscope SZX16 (Olympus) was used for Figs 2A, 3A, 3B, 5A, 5B, 5E, 5F, and Supplementary Fig. 6A. Confocal microscopes Zeiss LSM510 META/Axiovert 200 (Olympus) was used for Figs 1D, 2B, 2E, Supplementary Fig. 3A and Supplementary Movie 1-3. FV3000 (Olympus) was used for Figs 4A and 4E, while FV4000 (Olympus) was used for Fig. 4F. BX51 (Olympus) was used for Figs 2C, 2D and Supplementary Figs 3B and 3C. SU8220 (Hitachi High-Tech) was used for Figs 2C, 2D and Supplementary Figs 3B and 3C. IX73

inverted microscope (Olympus) was used for Supplementary Figs 4A, 4C, and 4D, and Supplementary Movie 4 and 5. The images were captured and processed using Olympus cellSens software.

Fluorescence stereo microscope SZX16 is equipped with a digital charge-coupled device camera (Olympus DP80), a GFP filter (Olympus SZX2-FGFPHQ, Ex 460-480nm / Em 495-540 nm), a YFP filter (Olympus SZX2-FYFPHQ, Ex 490-500nm / Em 515-560 nm) and an RFP filter (Olympus SZX2-FRFP2, Ex 540-580nm / Em 610- nm).

Zeiss LSM510 META/Axiovert 200 is equipped with a Plan Fluor 100×/1.45 NA oil objective. GFP and mVenus fluorescence were obtained with a multiline 488 argon laser for excitation and a 505–550 nm filter for emission. An HFT 405/514 beam splitter was used as a connecting filter. The transmitted light image was provided using Nomarski difference interference contrast (DIC).

FV3000 is equipped with six solid-state diode lasers (405 nm, 445 nm, 488 nm, 514 nm, 561 nm, and 640 nm)It uses Olympus objectives including PLAPON 40x (1.4 NA, oil-dipped) and UPLSAPO 100x (1.35 NA, Si oil-dipped). For image detection, the FV3000 is equipped with high-sensitivity GaAsP detectors, enabling the detection of faint fluorescence signals with high signal-to-noise ratios. GFP fluorescence was obtained with a 488-laser excitation with all diode lasers and LED illumination and a 500–600 nm filter for emission. The transmitted light image was provided using Nomarski difference interference contrast (DIC).

FV4000 comes with ten laser lines ranging from 405 nm to 785 nm and is equipped with Olympus objectives including UPLSAPO 100x (1.45 NA, Si oil-dipped). For image detection, the FV4000 uses the SilVIR detector, which combines a silicon photomultiplier (SiPM) with advanced signal processing. GFP fluorescence was obtained with a 488-laser excitation with all diode lasers and LED illumination and a 500–540 nm filter for emission. The transmitted light image was provided using Nomarski difference interference contrast (DIC).

BX51 is equipped with a digital charge-coupled device camera (Olympus DP71). It uses Olympus objectives including UPlanSApo 4x/0.16, UPlanSApo 10x/0.40, UPlanSApo 20x/0.75 and UPlanSApo x40/0.95. The mirror unit for GFP is U-MNIBA3 (Olympus, Ex 470-495nm / Em 510-550 nm). SU8220 was used at 15 or 20 kV accelerating voltage. The scanning mode for high magnification is SE(UL).

IX73 inverted microscope is equipped with a lens UPLAPO OI3 100x / 1.35 and flagellar length was measured by CellSens using the data taken as differential interference contrast (DIC) images.

Microscopic analysis of cell entry to a single pore of the stomata

Leaves were placed statically overnight on a bacterial solution containing hypho medium with no carbon source. The bacterial solution was prepared at OD_{600} 0.1. The leaves were then observed by confocal microscope FV3000 (Olympus) for quantitative analysis.

Microscopic analysis of the intracellular localization of GFP-MtpA

Cells cultured on hypho medium containing 0.5% methanol as a carbon source were used for microscopic observation or for preparation of a bacterial solution. Leaves were placed statically overnight on the bacterial solution containing hypho medium with no carbon source. The bacterial solution was prepared at OD_{600} 0.1. The leaves were then observed by confocal microscopes FV4000 (Olympus).

Flagella visualization

Living cells were stained for flagella visualization using the staining solution by Ryu[6] and a method similar to Heimbrook and colleagues[7]. Visualized flagella were investigated with an IX73 inverted microscope (Olympus) and the length of flagella was analyzed by cellSens.

Correlative light and electron microscopy (CLEM) analysis

Red perilla seeds were sterilized and treated with the cell suspension of strain OR01-GFP. They were sown onto Hoagland agar in a plant culture dish for growth in a chamber. One month after aseptic growth, aerial parts of the plant were harvested. Samples were fixed with a solution of 2% paraformaldehyde, 2.5% glutaraldehyde, 0.1 M phosphate buffer, pH 7.4 for 30 minutes at room temperature. Subsequently, these samples were used for CLEM analysis. After fixation, these samples were suspended and stored in phosphate-buffered saline. Subsequently, the samples were mounted on a glass slide and fluorescent and Bright-field images were acquired with BX51 (Olympus). After washing in 0.1 M phosphate buffer (pH7.4) three times, the

samples were frozen in liquid nitrogen for 2 minutes and freeze-dried overnight (Yamato Scientific Co., ltd. DC-56A). Then, the samples were mounted on SEM stubs using double-sided carbon tape and coated with 7 nm osmium in an HPC-1SW coater (Shinku Device). A scanning electron microscope (Hitachi High-Tech SU8220) was used for imaging according to the manufacturer's protocol. After image acquisition, images were merged using Photoshop 2024 (Adobe).

Colony formation analysis

After sterilization, as previously described[8], red perilla seeds were suspended in cells of strain OR01-GFP for 3 hours and sown onto Hoagland agar or vermiculite. Three to four months after aseptic growth, aerial parts of the plant were harvested. The collected samples were rinsed, suspended in sterile water and spread onto the hypho medium agar plate containing 0.5% methanol with kanamycin (20 μg/mL) as needed. To visualize colonies, the FAS-Digi imaging system (NIPPON Genetics) was employed.

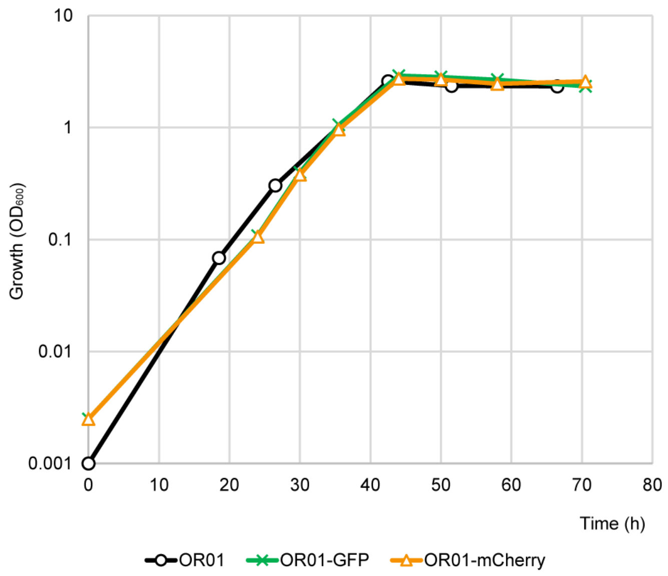

Supplementary Fig. 1. Growth of strain OR01, strain OR01-GFP and strain OR01-mCherry.

These strains were grown on hypho medium containing 0.5% methanol. Symbols: ○; strain OR01, ×; strain OR01-GFP, △; strain OR01-mCherry.

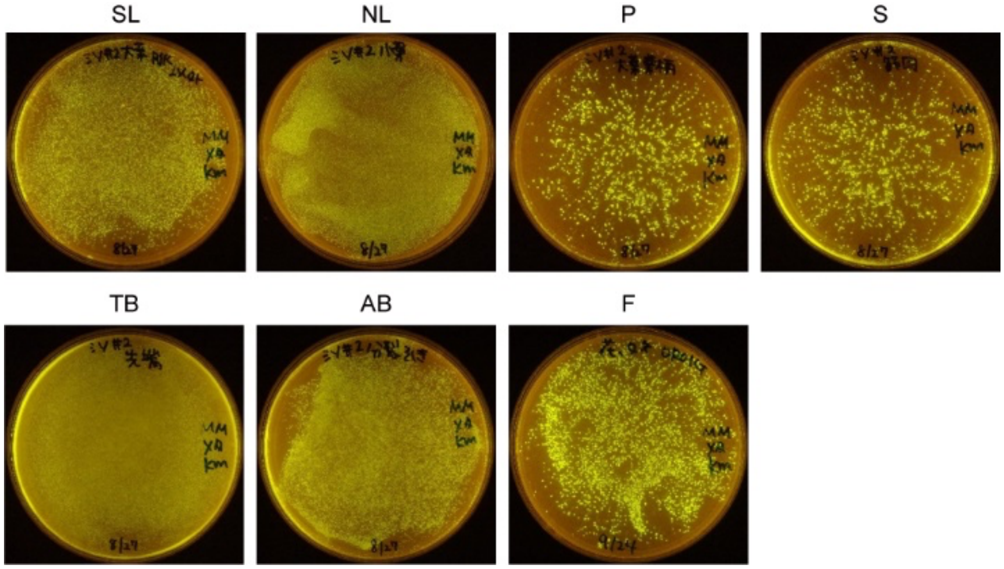

Supplementary Fig. 2. Colony formation activity of strain OR01-GFP collected from various parts of red perilla.

Plant samples were harvested and suspended in sterile water. The bacterial cell suspension was spread onto hypho medium agar plates supplemented with 0.5% methanol. Strain OR01-GFP was detected by FAS-Digi imaging system after 2–3 days.

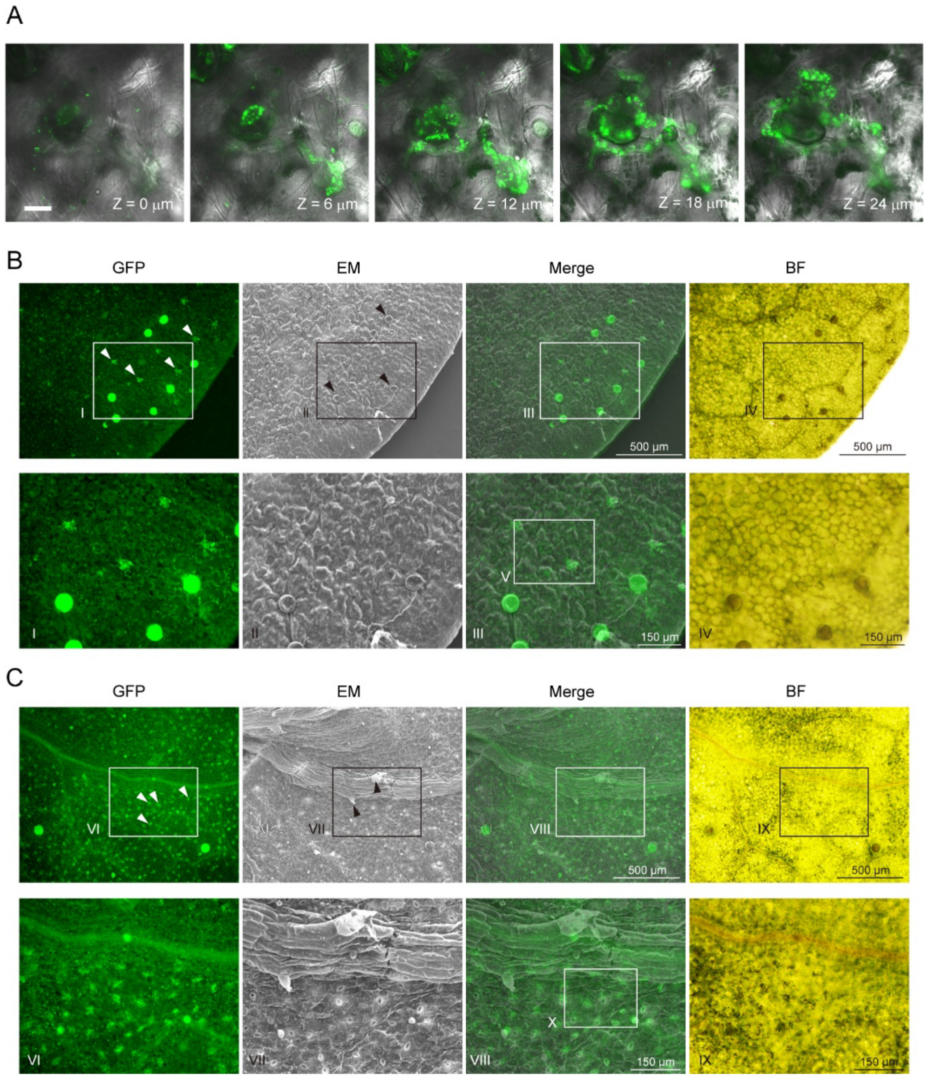

Supplementary Fig. 3. CLEM analysis of strain OR01-GFP colonizing red perilla leaves.
A Z-stack images of stomata with strain OR01-GFP taken by a confocal fluorescence microscope (see also Supplementary Movie 1). Every next image from the left to the right was taken at 6 µm deeper location compared to the previous one. Bar, 20 µm.
B CLEM images of strain OR01-GFP at a low magnification with a focus on the trichome (upper panels). GFP fluorescence images (left) and EM images (second from the left) were used for merged CLEM images (second from the right). Bright-field (BF) image is also shown (right). The images highlighted with squares (I), (II), (III) and (IV) were magnified (lower panels). The highlighted square (V) was the merged image used for Fig. 2C (upper panel). White arrows in the GFP image are clumps of strain OR01-GFP. Black arrow in the EM image a glandular trichome. Bars show the indicated lengths.
C CLEM images of strain OR01-GFP at a low magnification with a focus on the stomata (upper panels). GFP fluorescence images (left) and EM images (second from the left) were used for merged CLEM images (second from the right). Bright-field (BF) image is also displayed (right). The images highlighted with squares (VI), (VII), (VIII) and (IX) were magnified (lower panels). The highlighted square (X) was the merged image used for Fig. 2D (upper panel). White arrows in the GFP image shows bacteria colonizing around the stomata. Black arrow in the EM image is a trichome on the vein. Bars show the indicated lengths.

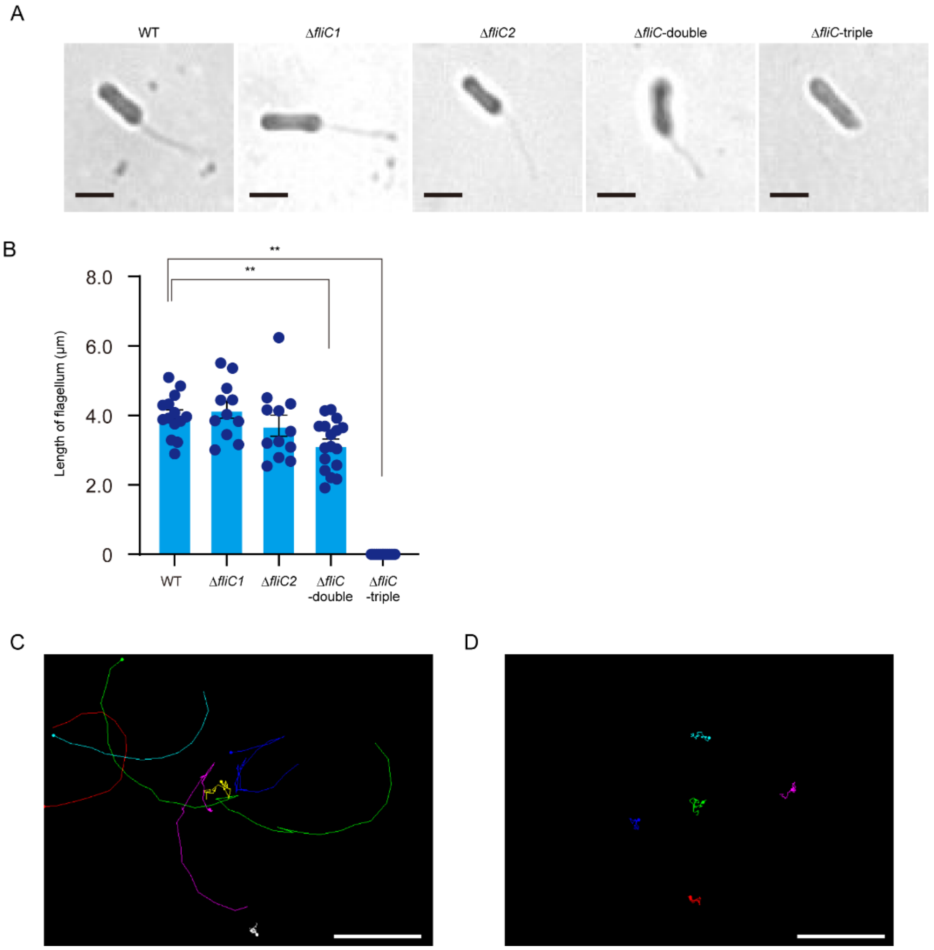

Supplementary Fig. 4. Deletion of *fliC* genes led to the loss of flagella.

A Inverted microscopic images of wild-type (WT), Δ*fliC1*, Δ*fliC2*, Δ*fliC1*Δ*fliC2* (Δ*fliC*-double) and Δ*fliC1*Δ*fliC2*Δ*fliC3* (Δ*fliC*-triple) cells of strain OR01. Flagella were visualized with *Ryu* stain, but could not be observed in strain Δ*fliC*-triple. Bar, 2 µm.

B Quantitation of the flagellum length measured from images taken by inverted microscope shown in (**A**). Values are indicated as the number of cells per mg of plant sample and are shown as the mean ± s.e.m. of a minimum of 11cells analyzed. Asterisks indicate the level of statistical significance between wild-type (WT) cells and Δ*fliC*-double cells, and WT cells and Δ*fliC*-triple cells: ** $p < 0.01$.

C-D The representative image plots of cell movement. (**C**) Single-cell tracking was analyzed by Manual Tracking plug-in for ImageJ. Plots were created with WT cells during the microscopic observation for 5.8 seconds. The period from 0.68 seconds to 6.48 seconds in Supplementary Movie 4 is shown in this figure. Seven cells were monitored. (**D**) Plots were created with Δ*fliC*-triple cells during the microscopic observation for 5.8 seconds. The period from 0.68 seconds to 6.48 seconds in Supplementary Movie 5 is shown in this figure. Five cells were monitored. Bar, 20 µm.

A

```
Strain_OR01_MtpA      MFSLRRQRHVSDAVATPVITPAPSTIPAAALIVEPLRPEPES---------------TVD
MaMtpA                ---MFRSRHAPAPVPVETPEMVEERLAGLVERAGASGSDLGSGRLARSLGQLVGRLQVAA
                         : *.**.. .*.. .   . . :.. .  . .  .:  *               ..

Strain_OR01_MtpA      REWLSLLARLSASASDAGTSIGWMTHDASGTAEQARLIAAASEELAATTGEIAARSSSAA
MaMtpA                AADLGSTARVAAEASEAATVLGWMTHDASEIAGQTRAMAAAVEEVAASTRELAGRSQASA
                         *.  **::*.**:*.* :********  * *:* :*** **:**:* *:*.**.::*

Strain_OR01_MtpA      ETAETARAGIVTCVGDLHRATEGMRGIEEGTEEIGSRLDSFSTAALRIEEMAGTIAAISA
MaMtpA                EVAERASGGIASCAADMREASSTMAAIETHAGQIEQRLTGFESAALQIQEMAGTIEAISS
                      *.** * .**.:*..*::.*:. * .**  : :* .** .*.:***:*:****** ***:

Strain_OR01_MtpA      QTNLLALNATIEAARAGEAGRGFAVVAAEVKTLSAQTAKATDEIRARVSGLREEMAAMQA
MaMtpA                QTNLLALNATIEAARAGEAGRGFAVVAAEVKQLSGQTARATEQIRGRLAVLLQELAAIQA
                      ******************************* **.***:**::**.*:: * :*:**:**

Strain_OR01_MtpA      AVTRSRNAVEAGASAMMRANARVEAESGAVANVAAQMREASEILGQQMQATGDIAQNVGR
MaMtpA                AVAESRHAVASGTAAVARVEARVVQEGDAVARSAEGIRALAEVLGQQEAATAEISSGVQQ
                      **:.**:** :*::*: *.:***  *..***. *  :*  :*:****  **.:*:..* :

Strain_OR01_MtpA      IAEGTDKARREIGDALAQLSRIEDLGRTLLDR-QIGTSADQQVIRLSADCAAWRRAIAAT
MaMtpA                VAGKAQKTSDEIKGLMAILVRAETKAQEVLDAGATRDLPGYRLLRLPADMGMWKRRLAAA
                      :*  ::*:  ** . :* * * *  .: :**       .. :::**.** . *:* :**:

Strain_OR01_MtpA      LVGMRST----------DTPPEGIPADSTRAAAIERPLAQAREQAAALIRHVRSQAWDQA
MaMtpA                LVGLGQASAAVPAFATGDAAELGLDASHPAAPQIVAACTEARRHAAAMVEALGAGAWDKA
                      ***: .:          *:.  *: *. . *. *  . ::**.:***::. : : ***:*

Strain_OR01_MtpA      TTAFVAFEAAVKEARSAIEA----
MaMtpA                IPAFQAFEAAAKTLVTAAEGATRH
                       .** *****.*   :* *.
```

B

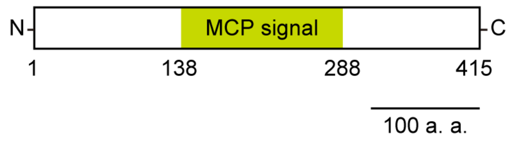

Supplementary Fig. 5. Comparison of the amino acid sequence of MtpA.
A Alignment of MtpA in strain OR01 (Strain_OR01_MtpA) with MaMtpA in strain 22A. MtpA in strain OR01 contains a 1242-bp ORF encoding a 414-amino acid protein. **B** Schematic diagram of MtpA structure from strain OR01. The conserved motifs were analyzed by GenomeNet MOTIF Search (https://www.genome.jp/tools/motif/), as described previously[3]. Methyl-accepting chemotaxis protein (MCP) signaling domain, demonstrated as MCP signal, is identified. Bar, 100 amino acids.

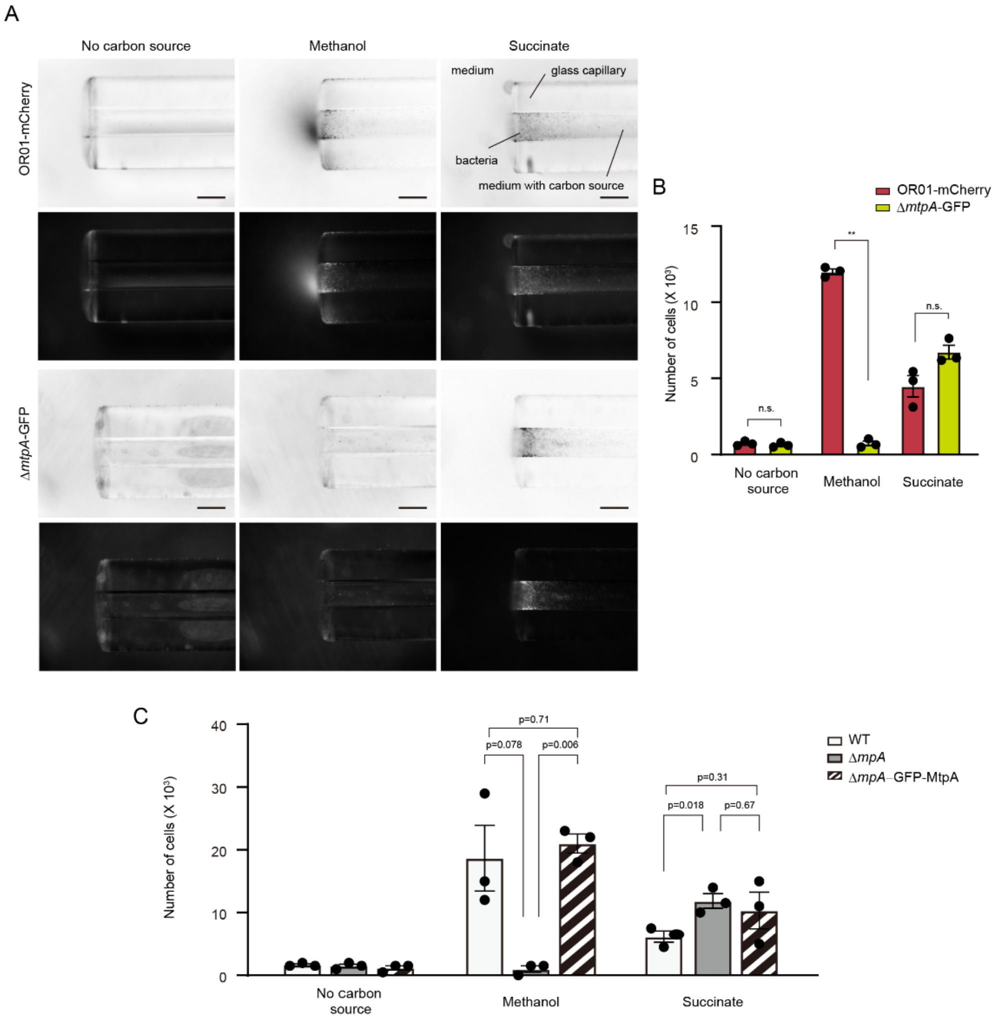

Supplementary Fig. 6. Deletion of *mtpA* gene led to a significant reduction in methylotaxis.
A Capillary assay for chemotactic response of strain OR01-mCherry and strain Δ*mtpA*-GFP. These strains were placed close to the glass capillaries between which a hypho medium supplemented with 0.05% methanol or 0.05% succinate. Hypho medium without any carbon source was used as a control. Outside of the capillaries were filled with a hypho medium without any carbon source. Images were taken by stereo microscope. Original pictures of capillary assays with strain OR01-mCherry and strain Δ*mtpA*-GFP are shown in the second and fourth rows, respectively. Exposure time for OR01-mCherry; No carbon source for 31s, methanol for 11.7 s, and succinate for 31s. Exposure time for Δ*mtpA*-GFP; No carbon source for 60 s, methanol for 60 s, succinate for 12.3 s. Bar, 200 µm.
B FCM-based quantification of cell populations of strain OR01-mCherry and strain Δ*mtpA*-GFP in capillaries used in **A**. Values are indicated as the number of cells per capillary and are shown as mean ± s.e.m. of analyses with three distinct capillaries. Asterisks indicate the level of statistical significance: * $p < 0.05$. Not significant: n.s.
C Complementation analysis of methylotaxis using WT strain, strain Δ*mtpA* and strain Δ*mtpA*-GFP-MtpA in hypho medium supplemented with 0.05% methanol or 0.05% succinate. Cells in each of the capillaries were spread onto hypho medium agar plates supplemented with 0.5% methanol. After 2–3 days when colonies appeared, the cell number was investigated by FCM. Values are indicated as the number of cells per capillary and are shown as mean ± s.e.m. of analyses with three distinct capillaries. Statistical significance is shown as p values.

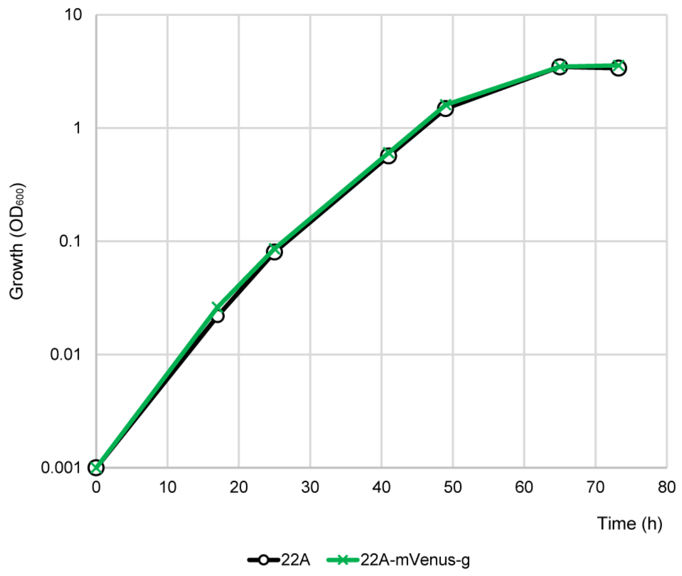

Supplementary Fig. 7. Growth of strain 22A and strain 22A-mVenus-g.
These strains were grown on hypho medium containing 0.5% methanol. Symbols: ○; strain 22A, ×; strain 22A-mVenusg.

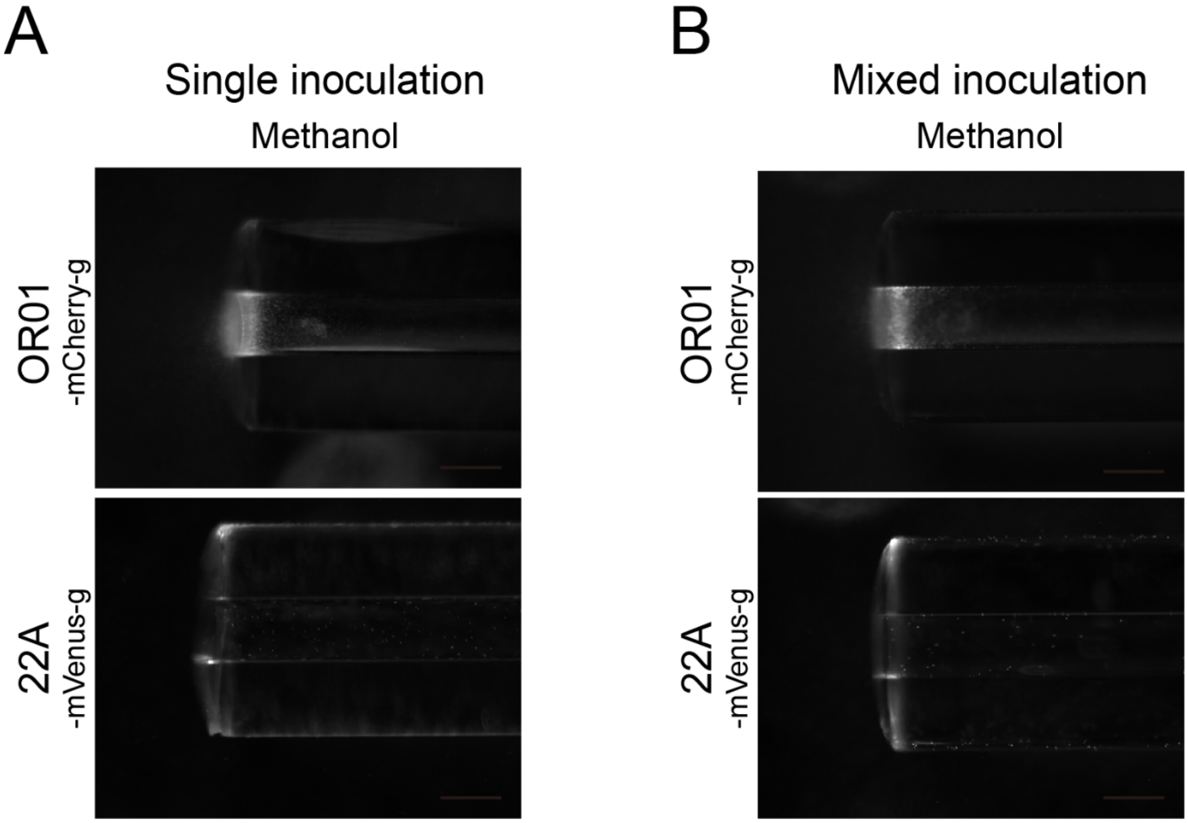

Supplementary Fig. 8. Capillary assay for methylotaxis of strain OR01-mCherry-g and strain 22A-mVenus-g.

A-B Original pictures of Figs 5E and 5F, respectively. Details of the experimental conditions are described in the legends to Figs 5E and 5F. **A**, Exposure time; OR01-mCherry-g for 2.9 s and 22A-mVenus-g for 10.3s. **B**, Exposure time; OR01-mCherry-g for 2.3 s and 22A-mVenus-g for 15.3s.

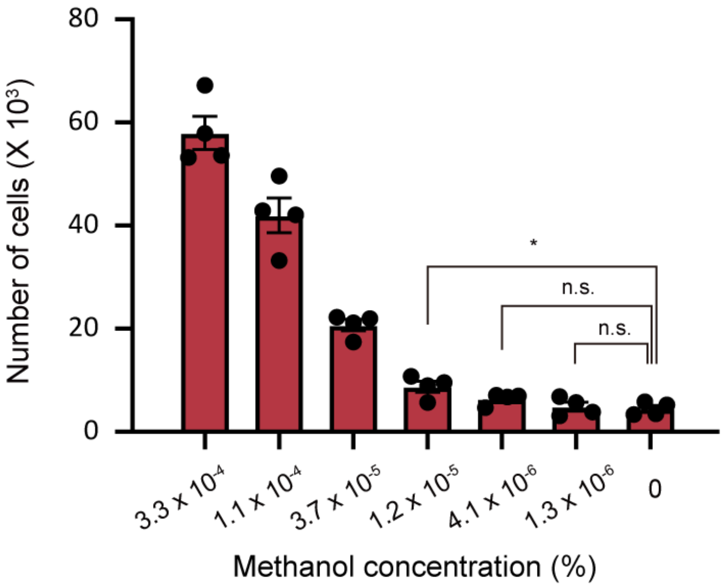

Supplementary Fig. 9. Capillary assay for chemotactic response of strain OR01-mCherry. Strain OR01-mCherry was placed close to the capillaries that contained a hypho medium supplemented with methanol at the indicated concentration and incubated at 28°C for 24 hours. Cell populations of strain OR01-mCherry in capillaries were quantified by FCM. Values are indicated as the number of cells per capillary and are shown as mean ± s.e.m. of analyses with four distinct capillaries. Asterisks indicate level of statistical significance: * $p < 0.05$. Not significant: n.s.

Supplementary Movie 1. Z-stack videography of strain OR01-GFP on the red perilla leaf surface with a focus on stomata.
Z-stack images of stomata with strain OR01-GFP taken by a confocal fluorescence microscope (see also Supplementary Fig. 3A). Five images were taken with 6 µm depth in the z-axis direction.

Supplementary Movie 2. Videography of strain OR01-GFP entering into the stomatal cavity.
Time-lapse images of strain OR01-GFP entering into the stomata cavity (see also Fig. 2E). The video was taken by a confocal microscope and recorded for 77 seconds at 1x speed. Bar, 20 µm.

Supplementary Movie 3. Videography of strain HST08-GFP not entering into the stomatal cavity.
Time-lapse images of *Escherichia coli* HST08 expressing GFP not entering into the stomata cavity. The video was taken by a confocal microscope and recorded for 24 seconds at 1x speed. Bar, 20 µm.

Supplementary Movie 4. Motility analysis of wild-type cells.
Videography of wild-type (WT) cells grown on hypho medium containing 0.5% methanol and transferred to slide glasses for analysis (see also Supplementary Fig. 4C). The videos were taken for about 10 seconds. Bar, 20 µm.

Supplementary Movie 5. Motility analysis of Δ*fliC*-triple cells.
Videography of Δ*fliC*-triple cells grown on hypho medium containing 0.5% methanol and transferred to slide glasses for analysis (see also Supplementary Fig. 4D). The videos were taken for about 10 seconds. Bar, 20 µm.

Supplementary Table 1: Bacterial strains used in this study

Strain	Genotype	Reference
***Methylobacterium* sp. strain OR01**		
OR01	Wild-type	Mizuno *et al.* 2013[8]
OR01-GFP	OR01::(pCM802-GFP, Km^r)	This study
OR01-mCherry	OR01::(pCM802-mCherry, Km^r)	This study
OR01-mCherry-g	OR01 locus 402468-403563::(pCM1684, Km^r)	This study
Δ*fliC1*	OR01Δ*fliC1*	This study
Δ*fliC2*	OR01Δ*fliC2*	This study
Δ*fliC*-double	OR01Δ*fliC1*Δ*fliC2*	This study
Δ*fliC*-triple	OR01Δ*fliC1*Δ*fliC2*Δ*fliC3*	This study
Δ*fliC*-triple-GFP	Δ*fliC1*Δ*fliC2*Δ*fliC3*::(pCM802-GFP, Km^r)	This study
Δ*mtpA*-GFP	Δ*mtpA*::(pCM802-GFP, Km^r)	This study
Δ*mtpA*-GFP-MtpA	Δ*mtpA*::(pCM802-GFP-mtpA, Km^r)	This study
***M. aquaticum* strain 22A**		
22A	Wild-type	Tani *et al.* 2015[9]
22A-mVenus-g	22A locus 1717648-1719862::(pCM1685, Km^r)	This study
***Escherichia coli* HST08**		
HST08-GFP	HST08::(pCM802-GFP, Km^r)	This study

Supplementary Table 2: Composition of hypho medium

Reagent	Final concentration (mg/L)
K_2HPO_4	2530
NaH_2PO_4	2250
$(NH_4)_2SO_4$	500
$MgSO_4$ 7 H_2O	200
EDTA 2Na	12.74
$ZnSO_4$ $7H_2O$	4.4
$CaCl_2$ $2H_2O$	1.466
$MnCl_2$ $4H_2O$	1.012
$FeSO_4$ $7H_2O$	0.998
$(NH_4)_6Mo_7O_{24}$ $4H_2O$	0.22
$CuSO_4$ $5H_2O$	0.314
$CoCl_2$ $6H_2O$	0.322

The following Vitamin mix was added to the medium, as necessary.

Reagent	Final concentration (μg/L)
Ca-pantothenate	400
Inositol	200
Niacin (nicotinic acid)	400
p-Aminobenzonate	200
Pyridoxine HCl	400
Biotin	2
Thiamin HCl	400

Supplementary Table 3: Plasmids used in this study

Plasmid	Description	Reference
**Fluorescent markers**		
pBluescript II SK(+)	Cloning vector, Ap^r	Stratagene Inc.
pDCG-1	Source of GFP, Ap^r Cm^r	Iguchi *et al.* 2013[1]
pMO149	Source of mCherry, Ap^r	Maeda *et al.* 2015[2]
pAT02-V	Source of mVenus, Ap^r	Tani *et al.* 2023[3]
pBS-P_{mxaF}	pBluescript II SK(+) carrying the *mxaF* promoter of *Methylobacterium* sp. strain OR01	This study
P_{mxaF}-GFP	pBS-P_{mxaF} expressing *GFP* under the *mxaF* promoter of *Methylobacterium* sp. strain OR01	This study
P_{mxaF}-mCherry	pBS-P_{mxaF} expressing *mCherry* under the *mxaF* promoter of *Methylobacterium* sp. strain OR01	This study
pCM80-Km	Expression vector carrying the *mxaF* promoter of *M. extorquens* strain AM1, Km^r	Orita *et al*. 2014[4]
pCM802-Km	pCM80-Km without carrying the *mxaF* promoter of *M. extorquens* strain AM1, Km^r	This study
pCM802-GFP	pCM802-Km expressing *GFP* under the *mxaF* promoter of *Methylobacterium* sp. strain OR01	This study
pCM802-mCherry	pCM802-Km expressing *mCherry* under the *mxaF* promoter of *Methylobacterium* sp. strain OR01	This study
pCM802-GFP-mtpA	pCM802-Km expressing *GFP-mtpA* under the *mtpA* promoter, Km^r	This study
pCM1682	Allelic exchange vector with *katA*::Km^r	Iguchi *et al.* 2018[5]
pCM1684	Allelic exchange vector with (genome region of strain OR01 at 402468-403563 locus)::Km^r, expressing *mCherry* under the *mxaF* promoter	This study
pCM1685	Allelic exchange vector with (genome region of strain 22A at 1717648-1719862 locus)::Km^r, expressing *Venus* under the *mxaF* promoter	This study
**Gene deletion**		
pK18mobsacB	Cloning vector, mob, sacB, Km^r	Iguchi *et al.* 2013[1]
pK18 fliC1	pK18 mobsacB harboring *fliC1* and the homologous fragments flanking *fliC1*	This study
pK18 ΔfliC1	pK18 mobsacB harboring the homologous fragments flanking *fliC1*	This study

pK18 fliC2	pK18 mobsacB harboring *fliC2* and the homologous fragments flanking *fliC2*	This study
pK18 ΔfliC2	pK18 mobsacB harboring the homologous fragments flanking *fliC2*	This study
pK18 ΔfliC3	pK18 mobsacB harboring the homologous fragments flanking *fliC3*	This study
pK18 mtpA	pK18 mobsacB harboring *mtpA* and the homologous fragments flanking *mtpA*	This study
pK18 ΔmtpA	pK18 mobsacB harboring the homologous fragments flanking *mtpA*	This study

Supplementary Table 4: Oligonucleotide primers used in this study

Primer names	Sequence (5'-3')
**Fluorescent markers**	
pCM80km-Re1800	GCGGTAATACGGTTATCCAC
pCM80km-Fw2471	CGCCAAGCTTGCATGCCTGC
PmxaF-Fw-KpnI	GGGGTACCACAGGTCGCCAGCGCCAGAA
PmxaF-Rv-HindIII	CCCAAGCTTCCTGCGTCTCCTCGCCGGAC
GFP-Fw-HindIII	CCCAAGCTTATGAGTAAAGGAGAAGAACT
GFP-Rv-PstI	AACTGCAGTTATTTGTAGAGCTCATCCA
mCherry-Fw-HindIII	CCCAAGCTTATGGTGAGCAAGGGCGAG
mCherry-Rv-BamHI	AAGGATCCAGGACTTGTACAGCTCG
1vector-Fw	TCATCTCAACATTATTTGAATACAGGGGCATCGACG
1vector-Rv	CAGAACCGGCCCACGCGA
3vector-Fw	CGCACAAGATGCCATGTATGG
3vector-Rv	CGCGAGGCGATATCGTCCATTC
OR01-2insertB-Fw	CGTGGGCCGGTTCTGCAACATCGTGCTCTACGCC
OR01-2insertB-Rv	ATGGCATCTTGTGCGGGTGTTGAACGAGAAGCCG
OR01-4insertB-Fw	CGATATCGCCTCGCGCTGCTCGGCATGGTGCTG
OR01-4insertB-Rv	ATAATGTTGAGATGAGCCGGTGCCTCAGGATCAG
PmxaF-KpnI-Fw	CGAGCTCCCGGGTACACAGGTCGCCAGCGCCAGAAA
mCherry-KpnI-Rv	TGCATGCCATGGTACCTTGTACAGCTCGTCCATGCCGCC
22A-2insertC-Fw	CGTGGGCCGGTTCTGCGCACATCTTCCGCCGTAC
22A-2insertC-Rv	ATGGCATCTTGTGCCGGAAGATGTGCGCCACCTCC
22A-4insertC-Fw	CGATATCGCCTCGCGGACGGCGGAAAGCGTGGAG
22A-4insertC-Rv	ATAATGTTGAGATGACGATATGCTGGTCGACATGC
EcoRI-PmxaF-Fw	TTGGTTGTAACACTGAATTCGCCGATGTCACCGTGCTG
GFP-EcoRI-Rv	CTTAAGCTCGAGGGCCCATGTAACTTGTACAGCTCGTCCA TGCCG
NB_PmtpA_Fw	AGGTCGACTCTAGAGGCCGAGACGGAGGATGCTC
NB_PmtpA+GFP_Rv	CTTTACTCATGCGGGACACTCCGATATCCG
NB_PmtpA+GFP_Fw	CCCGCATGAGTAAAGGAGAAGAACTTTTCACTGGAG
NB_GFP+mtpA_Rv	CTAAACATTTTGTAGAGCTCATCCATGCCATGTG
NB_GFP+mtpA_Fw	TCTACAAAATGTTTAGTTTGCGTCGGCAGC
NB_mtpA_Rv	CAGTGAATTCGAGCTTCAGGCCTCGATCGCCG
**Gene deletion**	
fliC1_up_Fw	GGGGATCCTCTAGAGTGGCGAGATCCATGGCTTC
fliC1_down_Rv	CCAGTGCCAAGCTTGTCCAGATCGGAGGTGCATC
Inv_fliC1_Fw	TCGCCGATCTGATTCAGGC
Inv_fliC1_Rv	GCGGCGTTGTTCGTAAACAG
fliC2_up_Fw	GGGGATCCTCTAGAGGATCTCTACGTCCGTCACACCG
fliC2_down_Rv	TGCCAAGCTTGCATGCCAATCCCCTGCTCAATGCAG
Inv_fliC2_Fw	GTTTGCATGAAAAGCGTCGG
Inv_fliC2_Rv	GAGCAGGTTCTGGCGTGTC
fliC3_up_Fw	GGGGATCCTCTAGAGTTGTCGTAATGCCATTTCGAG
fliC3_up_Rv	CGTCCGGTCAATGGTATCCTGCAGGGAGAGCAG

fliC3_down_Fw	ACCATTGACCGGACGGACGAGATG
fliC3_down_Rv	CCAGTGCCAAGCTTGGGCCTCAAAGATCCGGATCGCG
mtpA_up_Fw	TCGAGCTCGGTACCCCCCACCACGGAGTTCGAG
mtpA_ down_Rv	CTCTAGAGGATCCCCAGATCGAGTTCTGGCTGCTG
Inv_mtpA_Fw	CGTCGCGGGTCGTCTTAC
Inv_mtpA_down	CTCTAGAGGATCCCCAGATCGAGTTCTGGCTGCTG

Supplementary References

1. Iguchi H, Sato I, Yurimoto H *et al.* Stress resistance and C_1 metabolism involved in plant colonization of a methanotroph *Methylosinus* sp. B4S. *Arch Microbiol*. 2013;**195**:717-26. https://doi.org/10.1007/s00203-013-0922-6
2. Maeda Y, Oku M, Sakai Y. A defect of the vacuolar putative lipase Atg15 accelerates degradation of lipid droplets through lipolysis. *Autophagy*. 2015;**11**:1247-58. https://doi.org/10.1080/15548627.2015.1056969
3. Tani A, Masuda S, Fujitani Y *et al.* Metabolism-linked methylotaxis sensors responsible for plant colonization in *Methylobacterium aquaticum* strain 22A. *Front Microbiol*. 2023;**14**:1258452. https://doi.org/10.3389/fmicb.2023.1258452
4. Orita I, Nishikawa K, Nakamura S *et al.* Biosynthesis of polyhydroxyalkanoate copolymers from methanol by *Methylobacterium extorquens* AM1 and the engineered strains under cobalt-deficient conditions. *Appl Microbiol Biotechnol*. 2014;**98**:3715-25. https://doi.org/10.1007/s00253-013-5490-9
5. Iguchi H, Yoshida Y, Fujisawa K *et al.* KaiC family proteins integratively control temperature-dependent uv resistance in *Methylobacterium extorquens* AM1. *Environ Microbiol Rep*. 2018;**10**:634-43. https://doi.org/10.1111/1758-2229.12662
6. Ryu E. A simple method of staining bacterial flagella. *The Kitasato archives of experimental medicine*. 1937;**14** 218-219.
7. Heimbrook ME, Wang WL, Campbell G. Staining bacterial flagella easily. *J Clin Microbiol*. 1989;**27**:2612-5. https://doi.org/10.1128/jcm.27.11.2612-2615.1989
8. Mizuno M, Yurimoto H, Iguchi H *et al.* Dominant colonization and inheritance of *Methylobacterium* sp. Strain OR01 on perilla plants. *Biosci Biotechnol Biochem*. 2013;**77**:1533-8. https://doi.org/10.1271/bbb.130207
9. Tani A, Ogura Y, Hayashi T *et al.* Complete genome sequence of *Methylobacterium aquaticum* strain 22A, isolated from *Racomitrium aponicum* moss. *Genome Announc*. 2015;**3**:e00266-15. https://doi.org/doi:10.1128/genomea.00266-15
